# Genetic variation for adaptive traits is associated with polymorphic inversions in *Littorina saxatilis*

**DOI:** 10.1002/evl3.227

**Published:** 2021-05-07

**Authors:** Eva L. Koch, Hernán E. Morales, Jenny Larsson, Anja M. Westram, Rui Faria, Alan R. Lemmon, E. Moriarty Lemmon, Kerstin Johannesson, Roger K. Butlin

**Affiliations:** ^1^ Department of Animal and Plant Sciences University of Sheffield Sheffield United Kingdom; ^2^ Evolutionary Genetics Section Globe Institute University of Copenhagen Copenhagen Denmark; ^3^ IST Austria Klosterneuburg Austria; ^4^ CIBIO‐InBIO, Centro de Investigação em Biodiversidade e Recursos Genéticos Universidade do Porto Vairão Portugal; ^5^ Department of Scientific Computing Florida State University Tallahassee Florida FL 32306‐4120; ^6^ Department of Biological Science Florida State University Tallahassee Florida FL 32306‐4295; ^7^ Department of Marine Sciences University of Gothenburg Strömstad 45296 Sweden

**Keywords:** Divergence with gene flow, hybrid zone, QTL, recombination, structural variants, variance partitioning

## Abstract

Chromosomal inversions have long been recognized for their role in local adaptation. By suppressing recombination in heterozygous individuals, they can maintain coadapted gene complexes and protect them from homogenizing effects of gene flow. However, to fully understand their importance for local adaptation we need to know their influence on phenotypes under divergent selection. For this, the marine snail *Littorina saxatilis* provides an ideal study system. Divergent ecotypes adapted to wave action and crab predation occur in close proximity on intertidal shores with gene flow between them. Here, we used F2 individuals obtained from crosses between the ecotypes to test for associations between genomic regions and traits distinguishing the Crab‐/Wave‐adapted ecotypes including size, shape, shell thickness, and behavior. We show that most of these traits are influenced by two previously detected inversion regions that are divergent between ecotypes. We thus gain a better understanding of one important underlying mechanism responsible for the rapid and repeated formation of ecotypes: divergent selection acting on inversions. We also found that some inversions contributed to more than one trait suggesting that they may contain several loci involved in adaptation, consistent with the hypothesis that suppression of recombination within inversions facilitates differentiation in the presence of gene flow.

**Impact Statement**

Chromosomal inversion polymorphisms, segments of chromosomes that are flipped in orientation and occur in reversed order in some individuals, have long been recognized to play an important role in local adaptation. They can reduce recombination in heterozygous individuals and, thus, help to maintain sets of locally adapted alleles. In a wide range of organisms, populations adapted to different habitats differ in frequency of inversion arrangements. However, getting a full understanding of the importance of inversions for adaptation requires confirmation of their influence on traits under divergent selection. Here, we studied a marine snail, *L. saxatilis*, that has evolved ecotypes adapted to wave exposure or crab predation. These two types occur in close proximity on different parts of the shore. Gene flow between them exists in contact zones. However, they exhibit strong phenotypic divergence in several traits under habitat‐specific selection including size, shape, and behavior. We used crosses between these ecotypes to identify genomic regions that explain variation in these traits. We could show that previously detected inversion regions contribute to adaptive divergence. Some inversions influenced multiple traits suggesting that they contain sets of locally adaptive alleles. Our study also identified regions without known inversions that are important for phenotypic divergence. Thus, we provide a more complete overview of the importance of inversions in relation to the remaining genome.

## Introduction

Understanding the mechanisms that promote phenotypic diversification is of central interest in evolutionary biology. Some of the differences we observe in nature may not be caused by genetic divergence but by environmental effects. However, in many cases there is evidence for heritability of traits contributing to local adaptation (Hereford 2009), confirming that populations are genetically adapted to their native habitats (Savolainen et al. 2013). Genetic differentiation can even occur over very small geographical scales (Slatkin 1987) where differentially adapted populations are within dispersal range of each other. While some isolating mechanisms, like assortative mating (Servedio and Boughman 2017) or phenological differences, may contribute to keeping locally adapted entities apart, in many cases some level of gene flow exists (Lenormand 2002; Smadja and Butlin 2011), for instance, in contact zones with frequent hybridization (Wu et al. 2008; Harrison and Larson 2016; Schaefer et al. 2016; Chhatre et al. 2018). These examples have raised questions about the mechanisms maintaining and promoting genetic differentiation despite the homogenizing effects of gene flow (Felsenstein 1981; Pinho and Hey 2010).

Theoretical studies have found that certain genetic architectures favor local adaptation and protect locally advantageous alleles (Feder et al. 2012; Yeaman 2013; Rafajlović et al. 2016). Adaptation by fewer loci of large effect should proceed faster and be more resistant to gene flow under selection‐migration balance (Yeaman and Otto 2011; Yeaman and Whitlock 2011). Furthermore, it is expected that selection in different habitats is multivariate with many traits involved and potentially many contributing genetic loci. However, recurrent recombination is expected to break down advantageous allele combinations. If local adaptation is based on alleles at multiple loci, reduced recombination between them should be under positive selection in the presence of gene flow (Lenormand and Otto 2000) and can favor local adaptation (Kirkpatrick and Barton 2006). Thus, there should be selection for locally adapted alleles to be tightly linked, either by being physically close on the same chromosome or in regions of low recombination (Bürger and Akerman 2011; Yeaman and Whitlock 2011; Aeschbacher et al. 2017). In light of this, chromosomal inversions have received great interest for their potential role in local adaptation and speciation in the presence of ongoing gene flow (Feder and Nosil 2009; Smadja and Butlin 2011; Feder et al. 2012; Ravinet et al. 2017). Inversions are known to suppress recombination by impeding cross‐overs during meiosis in heterozygous individuals or leading to gametic imbalance and embryo abortion (Kirkpatrick 2010). Inversions can, thus, maintain sets of locally adapted alleles and prevent exchange with other genetic backgrounds, forming barriers to gene flow that might contribute to reproductive isolation (Rieseberg 2001; Navarro and Barton 2003; Faria et al. 2019b).

Over the past years there has been accumulating evidence that inversion polymorphisms contribute to local adaptation in a wide range of taxa (Hoffmann and Rieseberg 2008; Wellenreuther and Bernatchez 2018). Alternative arrangements often differ in frequencies between ecotypes (Twyford and Friedman 2015; Hanson et al. 2017; Christmas et al. 2019). Although these patterns are intriguing, and consistent with expectations from theory (Charlesworth and Barton 2018), the exact mechanisms are often not fully understood. Since selection acts on phenotypes, a full understanding of the specific role of chromosomal rearrangements for adaptation necessarily requires establishing the link between inversions and phenotypes under divergent selection in locally adapted populations. Empirical support for selection on inversions is often based on covariance with environmental variables, either by frequency fluctuations over seasons (Butlin and Day 1989; Ayala et al. 2011) or environmental clines (Ayala et al. 2014; Kapun et al. 2016a). Cases where these clines are replicated with consistent patterns across continents provide strong support (Kapun et al. 2016a; Mérot et al. 2018). However, confirming a direct causal influence is often challenging (Hoffmann et al. 2004; Kirkpatrick and Kern 2012). The exact features that make inversions important for local adaptation, suppression of recombination and maintenance of large regions in LD, also pose a substantial challenge for studying their content and identifying targets of selection. Using QTL and association mapping studies showed that they contribute to desiccation resistance in *Anopheles* (Ayala et al. 2019), fitness variation and divergence in monkeyflowers (Lowry and Willis 2010; Lee et al. 2016; Coughlan and Willis 2019), migratory behavior in cod (Sinclair‐Waters et al. 2018), mimicry in *Heliconius* butterflies (Joron et al. 2011), body size in *Drosophila* (Kapun et al. 2016b; Durmaz et al. 2018), and life‐history traits in seaweed flies (Butlin and Day 1985; Mérot et al. 2020). In most cases, the exact loci inside an inversion responsible for phenotypic variation could not be identified. Only a few studies have been successful in getting more insights, for example, finding linked color pattern loci within an inversion in *Heliconius* (Joron et al. 2011; Edelman et al. 2019) or ecologically important QTLs in *Boechera stricta* (Lee et al. 2017).

When studying inversion polymorphism in wild populations an additional challenge is imposed by potentially strong confounding effects of the environment on phenotypes. Most phenotypes are plastic, that is, influenced by the environment, which can lead to differences even in the absence of genetic differentiation. Furthermore, inversion frequency clines can also result from neutral, demographic processes and reflect patterns of colonization and range expansion (Klopfstein et al. 2006). Making robust conclusions about the role of inversions in local adaptation requires disentangling these effects from causal effects of inversions. It is therefore crucial to complement studies in the field with controlled lab experiments.

Here, we explored the role of inversions in phenotypic divergence in a well‐studied system, the marine snail *Littorina saxatilis*. This species has evolved divergent ecotypes associated with distinct shore habitats multiple times (Johannesson et al. 1993; Panova et al. 2006; Rolán‐Alvarez 2007; Butlin et al. 2014). Snails living on wave‐exposed rocks and those occurring in crab‐rich habitats differ in a range of traits including size, shell shape and behavior (Johannesson et al. 2010; Johannesson 2016). “Wave” snails are characterized by globular shells (Johannesson 1986) and a wide aperture, potentially adapted to prevent dislodgment by wave action (Le Pennec et al. 2017). In contrast, “Crab” snails are less exposed to wave action but experience predation pressure from crabs. They are two to three times larger and have thicker shells (even when controlled for size) with narrower apertures that impede crabs from either cracking the shell or pulling snails out (Johannesson 1986; Boulding et al. 2017). In addition, Wave snails are bolder, that is, more anxious to crawl out and remain attached to the surface, while Crab snails are wary and stay longer inside their shell after disturbance (Johannesson and Johannesson 1996). Phenotypes change across transition zones from one habitat to the next (Johannesson et al. 2010; Le Pennec et al. 2017; Westram et al. 2018). Previous studies have found them to persist, at least partially, in lab‐reared individuals (Johannesson and Johannesson 1996) suggesting a genetic basis. Although there is some evidence for assortative mating between ecotypes (Johannesson et al. 2008; Perini et al. 2020) ongoing gene flow between them is common (Panova et al. 2006; Westram et al. 2018). Recently, it was shown that the *L. saxatilis* genome contains multiple large inversions (regions of high linkage disequilibrium (LD); Faria et al. 2019a), with many of them showing frequency differences between the ecotypes and significant clinal patterns across the hybrid zones. Moreover, genetic differentiation between ecotypes has accumulated in genomic regions containing these putative inversions (Westram et al. 2018; Morales et al. 2019). However, the influence of these inversions on phenotypic divergence is mostly unknown.

To investigate the influence of inversions on local adaptation, we applied a powerful approach using more than 380 lab‐reared individuals resulting from crosses between the two divergent ecotypes. This strategy allowed us to remove confounding environmental effects and homogenize the genomic background of individuals. We used QTL mapping to test for associations between genomic regions and phenotypic traits distinguishing ecotypes. Furthermore, we applied variance partitioning across linkage groups to test whether chromosomes harboring inversions that differ in frequency between ecotypes contributed disproportionately to phenotypic variation. By using complementary approaches, we were able to capture different aspects of the genetic architecture of local adaptation beyond inversions and identify additional regions important for phenotypic divergence.

## Materials and Methods

### SAMPLE COLLECTION AND CROSSING

Crossing was performed between Crab and Wave ecotype individuals collected on the Swedish West Coast at Ängklåvebukten (58.8697°, 11.1197°), where both ecotypes occur in close proximity (see also Westram et al. 2018 ). The parental female snails were brought into the lab as juveniles and raised in isolation until maturity to prevent uncontrolled matings. The parental males were brought in as adults (more details in Supporting Information Appendix S1). Two virgin Crab‐females were crossed with two Wave‐males resulting in two F1‐families (Supporting information Appendix S1 Fig. I). Three males and three females of each F1‐family were then crossed reciprocally with an individual from the other family (see Supporting Information Appendix Fig. I). Unfortunately, genotypic data showed that offspring did not all belong to the expected families, potentially due to contamination from different tanks or nonvirginity of F1 females. To avoid parental misassignments, we evaluated relationships within the F2 and relationships to the presumed parents based on genomic data, following VanRaden (2008) as implemented in the Rpackage “AGHmatrix” (Amadeu et al. 2016), and adjusted the pedigree accordingly since misclassification of individuals as full sibs can lead to inflation of linkage maps (Supporting Information Appendix S1). This resulted in a total of 386 individuals divided into 13 F2‐families (eight full‐sib families and one half‐sib family that included five full‐sib groups, Supporting information Appendix S1 Table II) that were used for linkage map construction and phenotyping.

### GENOTYPING

DNA was extracted from a small piece of foot tissue using a CTAB protocol (Panova et al. 2016). We performed targeted resequencing at Florida State University's Center for Anchored Phylogenomics (www.anchoredphylogeny.com) as described in Faria et al. (2019a) and Westram et al. (2018), using a total of 25,000 (120 bp) enrichment probes. The majority of probes (20,000) were drawn from those that were informative in Westram et al. (2018). Novel probe regions (5000) were added to extend the existing linkage map, selecting one probe per contig from randomly drawn genomic contigs from the *L. saxatilis* reference genome as in Westram et al. (2018). Details of probes are provided in Supporting information Table S8. Raw reads were processed as described in Faria et al. (2019a); details in Supporting information Appendix S1.

### PHENOTYPES

**Figure 1:**
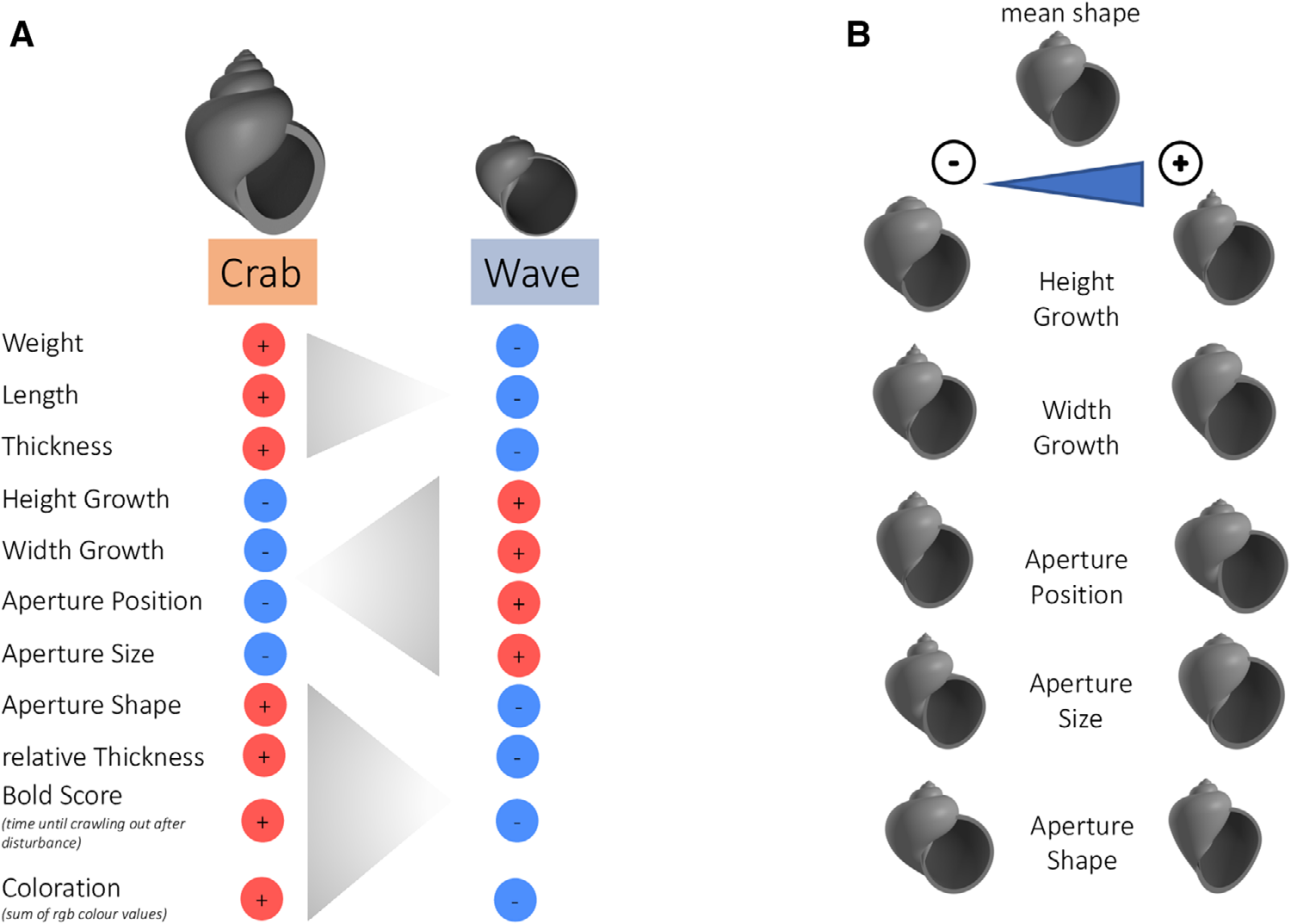
(A). Traits analyzed in this study and their association with ecotypes in the field. The Crab ecotype occurs in boulder fields and is exposed to Crab predation, whereas the Wave type can be found on rocky shores under wave exposure. Red + indicates that larger values are associated with the respective ecotype, blue – indicates smaller values. (B) Illustration of the different shape parameters analyzed in this study. Parameters are obtained based on a growth model (Larsson et al. 2020). The shape at the top represents the mean value of the whole F2 set. Each of the other shapes is varied for one parameter of interest, while all other parameters are held constant. The overall characteristic Crab and Wave shapes are shown in (A).

Phenotypes measured included weight, shell length, shell thickness (mean of three measurements per snail), relative thickness (size‐independent), shell shape, shell color, and boldness behavior that were previously found to differ between ecotypes (Johannesson et al. 2010). Size‐independent parameters for shell shape were obtained based on a growth model (Larsson et al. 2020). We included Height and Width growth, describing the shape of the shell, as well as the position (radial position in Larsson et al. 2020), size and shape (aperture extension in Larsson et al. 2020) of the aperture (Fig. 1). This previous study showed an association with environmental variables describing Crab/Wave habitats. Color was recorded as RAL categories (https://www.ralcolor.com) by visual matching to color cards by one of us (KJ). To obtain a continuous variable, we converted RAL categories to rgb‐color values (https://rgb.to/ral). For boldness behavior, snails were disturbed to induce retraction and time recorded until an individual crawled out (following Johannesson and Johannesson 1996). Observations were terminated after 15 min and individuals who had not emerged during that time were given a random value drawn from the tail of the distribution (log normal distribution of all observational times). Individual behaviors were tested three times on separate days and the average values (log of time) were used as Bold Score (lower values indicate bolder individuals, that is, less time until emergence). Measurements took place in 3 months (December 2014, March 2015, June 2015). Except for boldness and thickness, each phenotype was measured once (month of measurement was included in subsequent analyses). Sex of F2‐individuals was determined by dissection.

### LINKAGE MAP CONSTRUCTION

A linkage map was generated using LepMap3. We used 386 F2‐individuals (see Supporting Information Appendix S1) with 22,095 markers and combined all families for construction of one linkage map. The “ParentCall2” module was used (with options removeNonInformative = 1, halfsibs = 1) to calculate the most accurate parental genotype posteriors and to obtain missing parental information from offspring. We used the LepMap3 filtering module to remove markers with significant segregation distortion (dataTolerance = 0.01). Markers were grouped into Linkage Groups (LG) with the “SeparateChromosomes2” module, using a LOD score limit of 16 and sizeLimit = 100. We set lodLimit = 16 since this resulted in 17 LGs as was expected based on chromosome number (García‐Souto et al. 2018). Additional singular markers that could not be assigned in this step were subsequently added using the “joinSingles2all” (using lodLimit = 16, lodDifference = 2) function with 21 iterations. After assignments of markers to different LG, we ran the “OrderMarker2” module for each LG six times and selected the run with highest likelihood score. “OrderMarkers2” orders the markers within each LG by maximizing the likelihood of the data given the order. Markers not showing strong linkage with others that cannot be placed in the right order with certainty are placed to the ends of the LG. Therefore, we manually removed isolated markers causing long gaps (> 2cM) at the end of each LG. We then ran the “OrderMarker2” module again. The final map contained phased chromosomal marker data with imputed missing genotypes (using parameter outputPhasedData = 1, hyperPhaser = 1). Phased data were converted for QTL mapping using Lep‐MAP's map2genotypes.awk script. For subsequent QTL analysis, we averaged female‐ and male‐specific marker positions (option sexAveraged = 1 in the “OrderMarker2” module). To transfer the positions of the previously detected putative inversions, we used the positions of markers within these regions (see Faria et al. 2019a) that were in common with our new map. We used the minimum and maximum positions of these markers to define the boundaries of inverted regions in our map. Please note that this is only an approximation since some markers within the LD clusters of the previous map were not included in our data set (Supporting Information Table S1).

### QTL MAPPING

QTL mapping was performed in rQTL (Arends et al. 2010; Broman et al. 2019) using Haley‐Knott‐regression implemented in the “scan1” function. We included batch (month of measurement) and sex as covariates and ran QTL scans for all phenotypic traits. A genome‐wide significance threshold (0.95 quantile) was assessed by 10,000 permutations. Sex was analyzed as a binary trait (without covariates). Confidence intervals for the position of a QTL were inferred using the “lod_int” function. The three rgb values for color were analyzed as a multivariate trait using the Rpackages “ShapeQTL” (Navarro 2015) and rQTL (Arends et al. 2010; Broman et al. 2019). To confirm the colocalization of QTLs and inverted regions, we further tested the effect of inversion genotypes on phenotypes directly using linear mixed models (Rpackages “lme4” and “lmertest” [Bates et al. 2015; Kuznetsova et al. 2017]) with phenotype as response variable, sex, batch and inversion genotype as fixed effects and family as random effect. We used type I ANOVA tests to infer significance of inversion effects, that is, after correcting for sex and batch effects. Complete results are in Supporting Information Table S7 and Figure S5. Inversion genotypes of F1 parents and F2 progeny were inferred using clusters detected in a principal component analysis of SNPs in putatively inverted regions following an approach described in Faria et al. (2019a). For a detailed description, see Appendix S1. Genotypes of F1 parents and F2 individuals can be found in Supporting Information Table S2.

### CHROMOSOME PARTITIONING, REGIONAL HERITABILITY, AND GENETIC CORRELATIONS

QTL analysis may fail to find regions associated with phenotypic variation if a trait is highly polygenic and each locus has an effect below the detection threshold (Manolio et al. 2009; Rockman 2012). Quantitative genetic approaches that rely on comparing phenotypes of individuals with different degrees of relatedness can estimate overall heritability but do not give any information about the genetic loci involved. However, by using genomic markers and information on their position in a linkage map for calculating relationships it is possible to partition genetic variance across the genome and identify specific regions important for phenotypic variation. Regions can be whole chromosomes (Yang et al. 2010; Robinson et al. 2013) or smaller regions (Nagamine et al. 2012; Riggio et al. 2013). This approach was first applied successfully to estimate SNP‐based heritability for human height (Yang et al. 2010) but also to several natural populations (Robinson et al. 2013; Bérénos et al. 2015; Santure et al. 2015; Wenzel et al. 2015). By integrating variance due to rare and common alleles as well as many loci with only small effects into a single estimate of additive variance it potentially allows the identification of regions that cannot be detected by QTL analysis.

Relationships between individuals were based on genomic relationships inferred from genetic marker data using the same marker set as in the linkage map. Pairwise genomic relationship matrices were calculated using the method proposed by Yang et al. (2010) as implemented in the Rpackage “AGHmatrix.” Marker assignment to chromosomes (LGs) was based on the linkage map presented here. Chromosome partitioning was performed following the procedure described in Robinson et al. (2013). Briefly, relationships between individuals were estimated separately by using only genetic markers from a specific region and these different relationship matrices were then included in one model. We used linear mixed models (also known as “animal models”, see Kruuk 2004; Wilson et al. 2010) including the fixed effects of sex and batch (month of measurement) and random additive genetic effects which were divided into two parts, regional genomic and whole genomic additive genetic effects. For this, we calculated pairwise genomic relationship matrices using (1) all markers, (2) all markers excluding those of the focal LG, and (3) markers exclusively from the focal LG. First, we ran a model including the genomic relationships based on all markers (**model A**): Phenotype ∼ batch + sex + additive genetic effects (based on all markers). Next, we fitted three models for each LG:

**Model B**: including a relatedness matrix based on all markers except those on the focal LG: Phenotype ∼ batch + sex + additive genetic effects (all markers excluding focal LG)
**Model C**: including a relatedness matrix based on all markers except those on the focal LG and a second relatedness matrix using only markers from the focal LG. Phenotype ∼ batch + sex + additive genetic effects (all markers excluding focal LG) + additive genetic effects (markers of focal LG)
**Model D**: including relatedness using all markers plus a second relatedness matrix using only markers from the focal LG. Phenotype ∼ batch + sex + additive genetic effects (all markers) + additive genetic effects (markers of focal LG).

We then compared log likelihoods of the different models using likelihood‐ratio tests with one degree of freedom. We tested whether a LG explained significant variation in a trait by comparing the log likelihood of **model C** (genome‐wide excluding focal LG plus second relatedness matrix based on focal LG) with the log likelihood of the **model B** (genome‐wide excluding focal LG). Under a polygenic architecture with many contributing loci that are evenly distributed across the genome, we expect that variance explained increases with length of the LG. To identify certain LGs that deviate from this expectation and explain more variance than expected based on their length, we compared whether **model D** (genome‐wide plus focal LG) was significantly better than **model A** (genome‐wide model) (Robinson et al. 2013).

Next, we refined variance partitioning to smaller regions. Each chromosome was divided into regions of 200 adjacent markers based on our linkage map. Variance partitioning and significance assessment was conducted analogously to chromosome partitioning.

Pairwise genetic correlations were inferred using bivariate animal models using relationships estimated from all markers. Significance was assessed by likelihood‐ratio tests comparing the model with correlation to a model where the correlation was set to zero (Wilson et al. 2010).

Models were run in Asreml 3 (Gilmour et al. 2009) implemented in Asreml‐R (Butler et al. 2009).

## Results

### LINKAGE MAP

The final linkage map consisted of 18,949 markers across 17 LG with a total length of 1129.8 cM. Lengths of LGs ranged between 34.6 and 84.1 cM. These LGs corresponded well to those of the previously published map (Westram et al. 2018) (Supporting Information Table S3). LG numbering was adjusted to maintain consistency with previous *Littorina* studies. Consistent with the expectation of suppressed recombination when parents are heterozygous for alternative arrangements (see genotypes Table S2), we found that many markers within these regions (e.g., inversions 6.1/2 and inversion 14.1/2) shared the same position in our QTL map (Supporting information Fig. S1). However, in most cases markers from inversion regions showed some recombination and not all of them were in complete LD because some F1 parents were inversion homozygotes (Supporting information Fig. S1, see also Table S1). For other inversions (e.g., 1.1, 4.1, 9.1, 11.1), we expected little recombination suppression since most parental individuals were homozygous (Supporting information Table S2).

### QTL MAPPING: QTLS MAP TO INVERSION REGIONS

**Figure 2:**
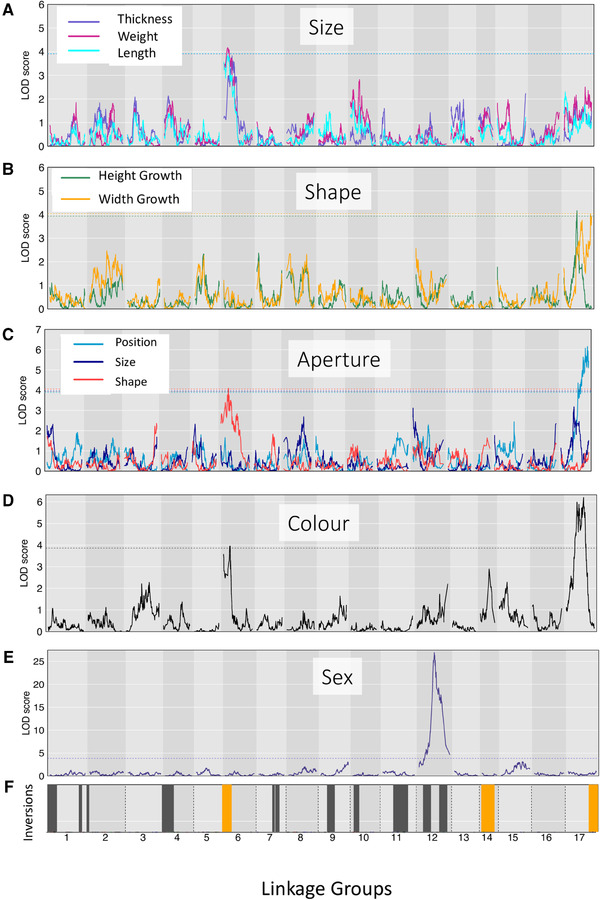
QTL scans for: weight, shell thickness and shell length (A); size independent parameters describing shell shape: Width and Height growth (B); for Aperture Size, Shape, and Position (C), shell color (rgb values) analyzed as a multivariate trait (D), and sex analyzed as binary trait (E). Dashed lines indicate genome wide significant thresholds (*P* = 0.05). Positions of putative inversion regions (± 2 cM) based on Faria et al. (2019a) (F). The positions are based on markers in common with the previous linkage map (based on a Crab/Crab cross). The exact positions of the inverted regions can thus only be approximated since markers at the utmost boundaries of the inversions were not always present in our map (see Supporting Information Table S1). Regions that showed an elevated proportion of non‐neutral SNPs based on cline analysis in the hybrid zone (Westram et al. 2018) and that overlap with inversions are indicated in orange.

Most of the studied traits showed suggestive peaks (LOD > 3) in the QTL analysis (Fig. 2, Supporting Information Table S4). We detected a significant QTL for weight (LOD = 4.17, *P* = 0.031) on LG 6. Shell thickness and length showed an almost identical pattern (Fig. 2A) but with slightly lower LOD (thickness 3.83, *P* = 0.06; length: 3.85, *P* = 0.08) that did not pass the significance threshold (*P* = 0.05). We found significant QTLs for the shape parameters: Height Growth (LOD = 4.16, *P* = 0.028) and Aperture Position (LOD = 6.16, *P* = 0.001) on LG 17, as well as for aperture shape on LG 6 (LOD = 4.36, *P* = 0.023; Fig. 2B, C). Other shape parameters showing suggestive peaks (LOD > 3) were Width Growth on LG 17 (LOD = 4.02, *P* = 0.053) and Aperture Size on LG 17 (LOD = 3.18, *P* = 0.21) and LG 12 (LOD = 3.12, *P* = 0.21). Color (based on rgb‐values) showed significant peaks on LG 6 and LG 17 (Fig. 2D). In contrast, no significant QTL could be detected for relative shell thickness (one suggestive peak on LG 2, LOD = 3.41, *P* = 0.14) or for Bold Score (Supporting information Fig. S2). However, we detected a highly significant QTL for sex on LG 12 (LOD = 26, *P* < 0.001, Fig. 2E).

**Figure 3:**
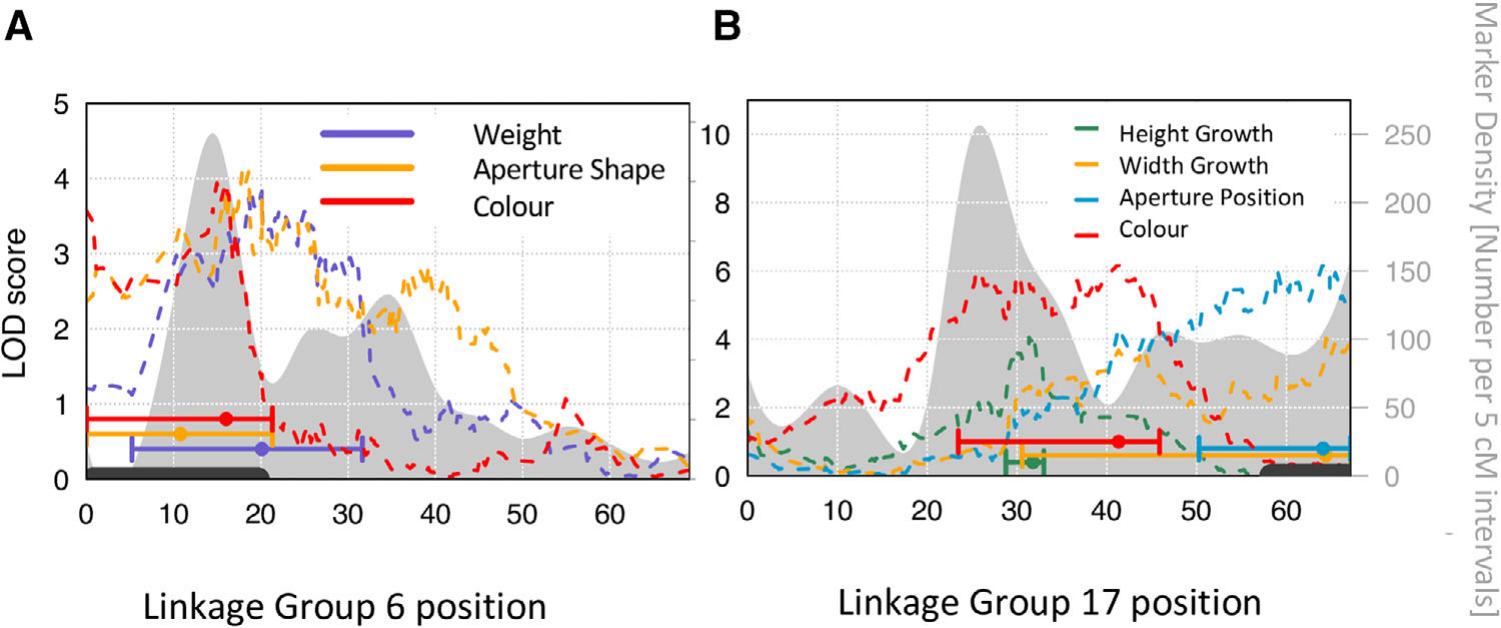
LOD scores for traits with significant QTLs (*P*‐value for Width Growth = 0.053) on linkage group 6 (A) and 17 (B) with the 95 % confidence interval (bars with CI) of their position. Position along the linkage group is given on the *x*‐axis and LOD scores (dashed lines) on the left *y*‐axis. Grey density plots give the marker density (number of markers per 5 cM intervals) along the linkage group (right *y*‐axis). Locations of inversions that were detected previously (Faria et al. 2019a) are shown by grey bars along the *x*‐axis. Regions of suppressed recombination with high marker density often coincide with previously described inversions. On both linkage groups, a clustering of QTLs within inverted regions is observed. On LG 17, we also see a cluster outside the inversion region consisting of QTLs for color, Height Growth, and Aperture Size (not significant, LOD = 3.12, *P* = 0.24).

All significant and most suggestive QTLs mapped to regions on LG 6 and LG 17. Closer inspection revealed that QTLs and their confidence intervals often overlapped with regions that were previously described as putative inversions (Supporting Information Table S1) and showed some suppression of recombination in our linkage map (Fig. 3, Supporting information Fig. S1). QTLs for weight, shell thickness, length, Aperture Shape, and color fell into the inversion region on LG 6 (Fig. 3A). QTLs for Width Growth and Aperture Position fell in the putative inversion region on LG 17. However, the QTL peak for Height Growth and color on LG 17 were outside the inversion (Fig. 3B). We tested the effects of inversions directly by genotyping F2 individuals for inversion arrangements. In general, we found the results of the QTL analysis to be confirmed: traits that showed significant QTL peaks in inversion regions (weight and Aperture Shape on LG 6 and Width Growth and Aperture Position on LG 17) were significantly influenced by the genotype of that respective inversion (Supporting information Table S7, Fig. S5). Interestingly, these results were also consistent with the localization of QTLs for Aperture Size, color, and Height Growth outside the inversion on LG 17 (Fig. 3B): No significant effect of inversion 17.1 on these traits could be detected (Supporting information Table S7). We could also see that other inversions, for example, inversion 1.1 and inversions on LG 12 (Supporting information Table S7) showed significant associations with phenotypes although no significant QTL peaks could be detected.

Variance explained by significant QTLs ranged from 4.3 to 7.4% (see also Supporting information Table S4). However, these estimates are upwardly biased since only significant QTLs are considered and effects of QTLs in low recombination regions are generally overestimated (Noor et al. 2001; Roesti 2018).

### LINKAGE GROUPS WITH INVERSIONS CONTRIBUTED DISPROPORTIONALLY BUT NONINVERTED REGIONS WERE IMPORTANT AS WELL

**Figure 4:**
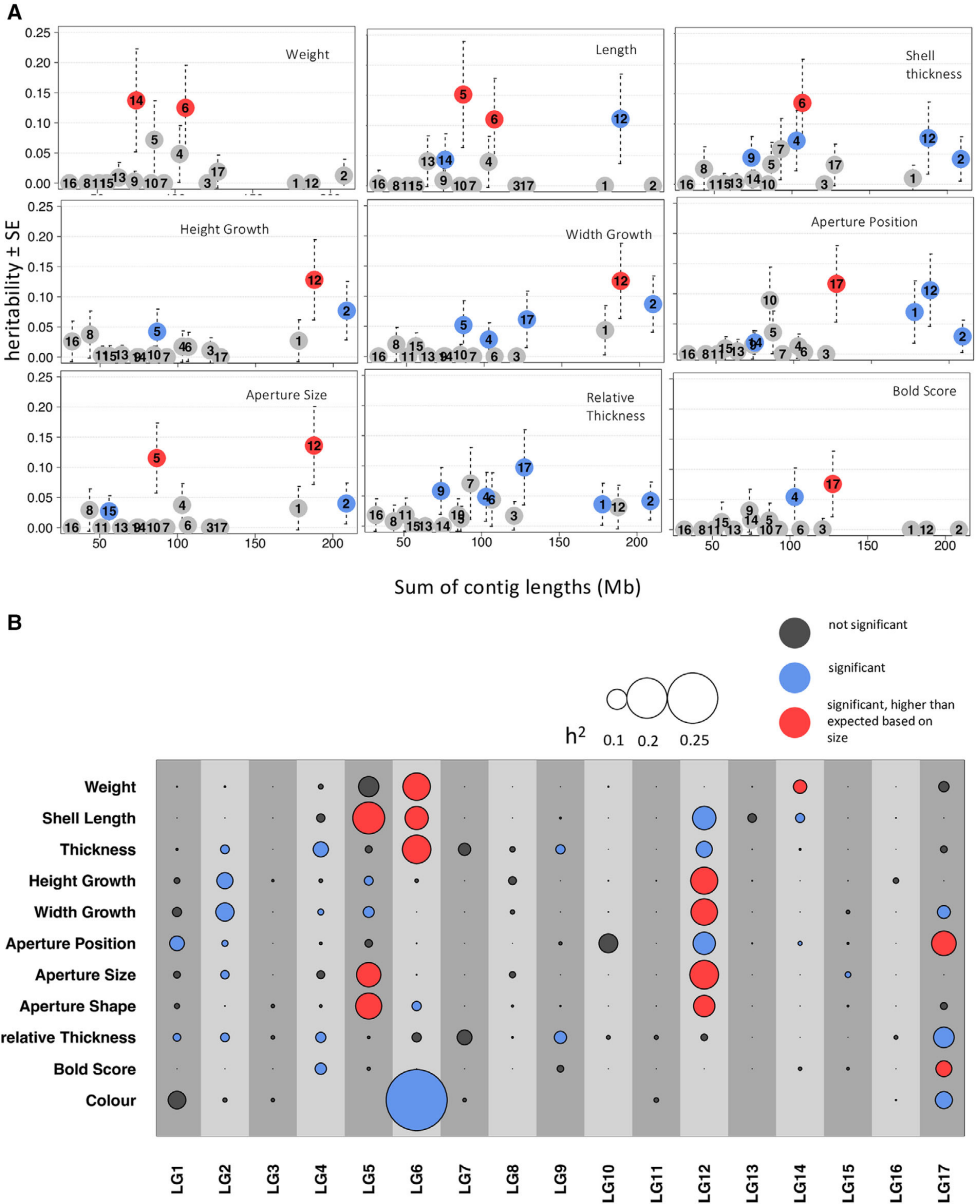
(A) Examples for proportion of phenotypic variance explained by different linkage groups (LG) ± standard error (SE) relative to sum of contig length that are assigned to each LG (proportional to chromosome length). If a trait is completely polygenic and loci are evenly distributed across chromosomes, a positive correlation between linkage group length and variance explained is expected. Deviations from polygenicity can be caused by large effect loci or clustering of loci. (B) Overview of LG‐specific heritability for all traits studied here. Circle size is proportional to LG‐specific heritability estimates. LGs explaining significant amounts of phenotypic variance are shown in blue; those explaining more phenotypic variance than expected based on their length in red.

Based on our chromosome partitioning analysis, several LGs contributed significantly to phenotypic variation (Supporting Information Table S5, Fig. 4), individual LGs explaining up to 16% of the total variance. Size‐related phenotypes (weight, shell length, thickness) were predominantly influenced by LG 6 whereas shape parameters (Height and Width Growth, aperture size, aperture position) were influenced by LG 5, 12, and 17 (Fig. 4). Summing point estimates of LG‐specific variances resulted in lower numbers than heritability (*h* ^2^) estimates obtained from a model that included markers from all LGs combined (Supporting information Table S5). Some inconsistencies can be expected given that *h* ^2^ estimates for each LG are surrounded by large standard errors (Fig. 4A). In some cases, statistical power for LG‐specific *h* ^2^ might have been too low resulting in zero estimates, which were probably underestimations.

**Figure 5:**
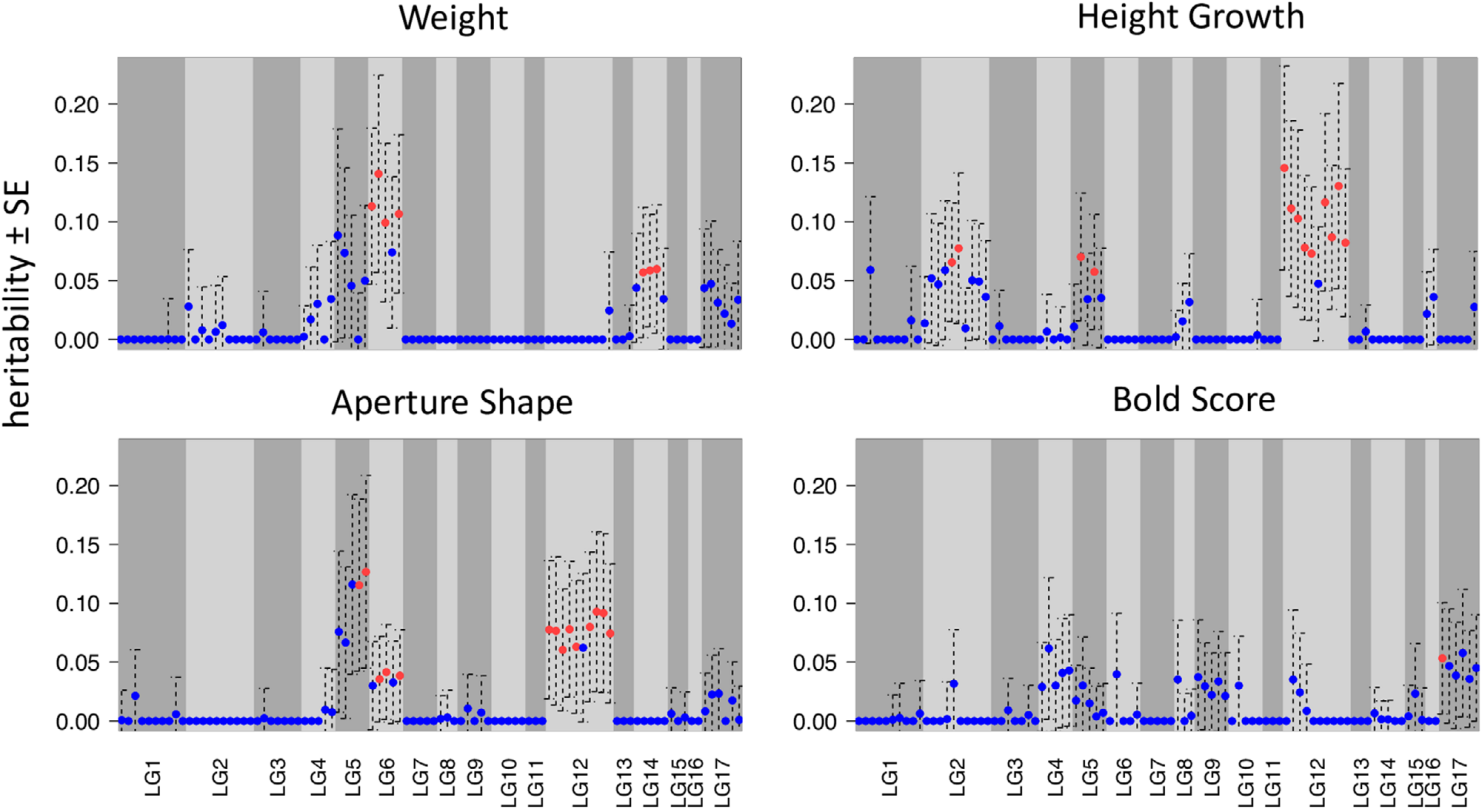
Examples for regional heritability ± standard error (SE) mapping of different traits. Each region consisted of 200 adjacent markers. Significant estimates are shown in red. Other traits can be found in Supporting Information Figure S3.

Results of variance partitioning and QTL analysis showed generally a good concordance. In most cases, LGs harboring QTLs were found to explain significant proportions of variance in the respective phenotype. LG 6 and 17, which showed a clustering of several QTLs, were also found to explain variance in more than one trait (Supporting information Table S5, Fig. 4B): LG 6 for weight, thickness, and shell length, LG 17 for Width Growth and aperture position. However, some LGs without any significant QTLs, not even suggestive peaks, explained high proportions of variance in several traits, namely LG 5 and 12. Consistent with this result, we found that inversions of LG 12 had significant influence on several traits (Supporting information Table S7) when we tested inversion genotype effects directly. Interestingly, LG 14, a strong candidate for being involved in ecotype divergence (Westram et al. 2018), but without a QTL peak in our analysis, was found to contribute to variation in weight (Fig. 4) and inversion 14.1/2 showed significant effects on size‐related traits thickness, weight, and shell length. Variance partitioning showed that several LGs contributed significantly more to trait variation than expected based on their length. These LGs included those with a clustering of QTLs in inverted regions (LG 6 and LG 17) but also LG 5 and LG 12 (Figs. 4, 5). Although larger LGs (e.g., LG 12, 2, 5) often contributed significantly to phenotypic variation, almost all traits deviated from the expected polygenic pattern (Fig. 4A, Supporting information Table S5).

### REGIONAL HERITABILITY MAPPING: ACCUMULATION OF OUTLIER REGIONS

Results of regional heritability mapping (RHM) were mainly consistent with variance partitioning across LGs. Significant regions were predominantly found on chromosomes that contributed significantly to trait variation and were almost always adjacent, with all regions of one LG often showing similar estimates, namely on inversion regions on LG 6, LG 12, LG 14, LG 17 (Fig. 5, Supporting information Fig. S3). Such a pattern is expected when closely related individuals are studied since there was not much opportunity for recombination to break down linked regions on the same chromosome, particularly in inverted regions where recombination is suppressed RHM estimates should be similar. However, RHM could in some cases provide additional insights. Linkage group 2 explained a significant amount of variance in Height and Width growth (Fig. 5, Supporting information Fig. S3). RHM suggested that the influence of LG 2 is not due to a cumulative effect of many loci that are evenly distributed across this LG but showed an accumulation of significant regions in the middle, outside known inversions (Fig. 5).

### GENETIC COVARIANCES AND ADAPTATION

**Figure 6:**
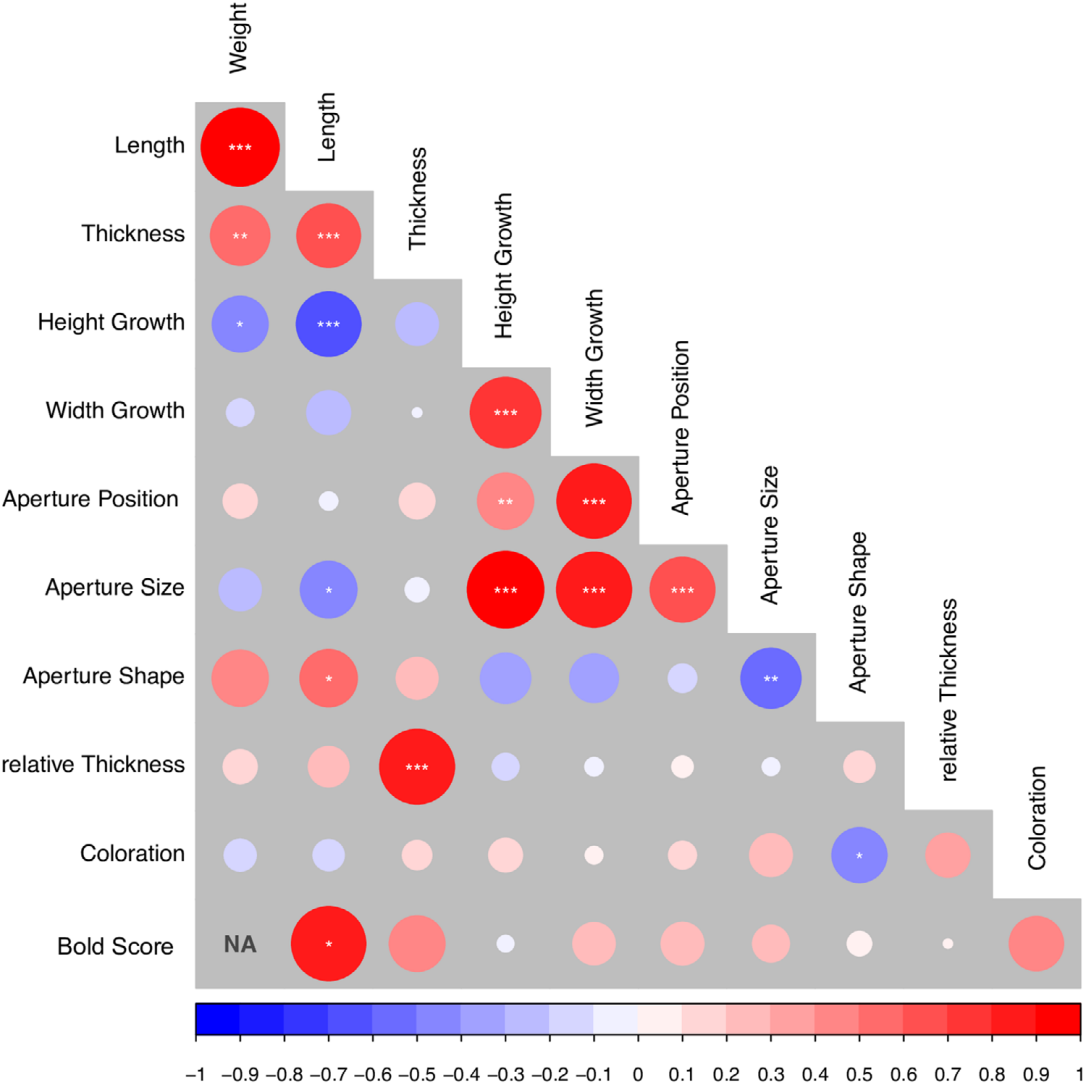
Genetic correlations between different traits estimated by bivariate animal models. Circle sizes are proportional to correlation coefficients. Significance was inferred from comparisons with models where correlation was set to zero using likelihood‐ratio tests. Due to lack of model convergence no estimates for correlation between weight and Bold Score can be reported. Significance: *P < 0.05; **P < 0.01; ***P < 0.001. Phenotypic correlations for the whole F2 as well as for each family separately are shown in Supporting Information Figure S4.

We found that most of the studied traits showed significant genetic correlations (Fig. 6, Supporting information Table S3). Traits falling into the same category form modules with high intercorrelation, for example, size‐related measures weight, thickness and length, as well as shape and aperture‐related measures, Width Growth, Height Growth and aperture variables. Interestingly, genetic correlations were almost always consistent with trait associations that characterize ecotypes in the field (see Fig. 1A, Johannesson et al. 2010; Larsson et al. 2020). Wave shape (large Height and Width growth) was genetically correlated with larger apertures and a smaller total size. In contrast, larger individuals tended to show smaller and narrower apertures (Fig. 1B), whereas smaller snails have larger and rounder apertures. We could also find genetic correlations between very different trait types. Bold Score showed a positive correlation with shell length (Fig. 6, Supporting information Table S6). Since time until coming out of the shell was measured, this means that larger individuals needed longer time until they crawled out of their shell after disturbance. Relative thickness and coloration (sum of rgb‐color values, i.e., lower values for darker shells) did not show significant correlations with other traits. However, the estimated correlation coefficients were mostly consistent with ecotype differences: positive correlations between relative thickness and size, Bold Score and coloration (bolder individuals have a darker shell), and Bold Score and relative thickness (bolder individuals have thinner shells). Phenotypic correlations based on all individuals (Supporting information Fig. S4A) as well as phenotypic correlations for each family separately (Supporting information Fig. S4B‐J) did not show strong differences.

## Discussion

This study contributes to our understanding of the role of inversion polymorphisms in local adaptation by confirming their influence on traits under divergent selection. Using lab‐reared F2 individuals from crosses between *L. saxatilis* ecotypes allowed us to avoid confounding environmental effects and enabled us to identify genomic regions important for phenotypic divergence between ecotypes. We show that traits that have diverged between ecotypes are significantly influenced by genomic regions previously described as putative inversions (Faria et al. 2019a). QTL analysis revealed a clustering of significant loci in these regions and we detected a significant association between inversion genotypes and traits. However, since power to detect candidate loci depends on LD between markers and causal loci, these regions are prone to exhibit significant QTLs. An approach combining QTL analysis with variance partitioning across chromosomes may, thus, help us to better evaluate the contribution of inversion regions compared to the remaining genome. Candidate LGs with inversions containing loci for ecotype divergence (based on genomic differentiation [Morales et al. 2019] or showing significant clines [Westram et al. 2018]) contributed disproportionately to phenotypic divergence. However, we also detected regions outside inversions that seem to be important for phenotypic variation. Notably, we found that phenotypic trait associations that characterize ecotypes in the field are genetically correlated and in many cases candidate LGs with inversions contributed significantly to more than one trait. Although exact insights into underlying mechanisms are not possible at present, this result suggests that inversions contain sets of coadapted alleles that facilitated the rapid and repeated formation of these ecotypes.

### INVERSIONS ARE INVOLVED IN LOCAL ADAPTATION AND UNDER DIVERGENT SELECTION

Previous studies have characterized phenotypic divergence between snails collected in the Crab and Wave habitats, including size, shape and behavioral differences, and found them to persist under lab conditions, at least in part (Johannesson et al. 2010; Johannesson 2016). In contrast, overall genetic differentiation between these ecotypes is low (Panova et al. 2006; Westram et al. 2018; Morales et al. 2019). However, a consistent pattern was an accumulation of outliers in putatively inverted regions on LGs 6, 14, and LG 17 at this particular Swedish site (Westram et al. 2018) and elevated divergence in these regions in several European populations (including Sweden, Spain, UK, and France) (Morales et al. 2019).

Genotyping the parental and F2 individuals confirmed that inversions were segregating. For two of these strong candidate regions (LG 6 and LG 17), we detected significant associations with several traits. Since selection acts on phenotypes, gaining a deeper understanding of the process leading to phenotypic divergence and local adaptation requires establishing the link between observed phenotypic and genetic differentiation. Our results, thus, complement these previous studies and confirm the role of inversions in ecotype divergence and local adaptation. They also add evidence that observed frequency clines of inversions across the habitat transition zone are not solely the product of neutral processes, for example, isolation by distance, genome‐wide barrier effects, or hitchhiking with a beneficial allele outside the inversion (Kirkpatrick and Barton 2006; Westram et al. 2018).

We found that LG 6 had a strong influence on all size‐related measures (weight, thickness, shell length). Size almost universally shows a positive correlation with reproductive output and thus often appears under positive selection (Blanckenhorn 2000). However, faster growth rate may trade‐off with reaching sexual maturity later. If mortality in the wave habitat is higher or higher for large individuals that get more easily dislodged, alleles promoting sexual maturity early but retarding growth might be under positive selection (Janson 1983). In contrast, Crab snails may be under selection to increase size rapidly to escape predation, with reproduction starting later (Boulding et al. 2017) resulting in Crab snails having higher growth rates (Janson 1982) and being two to three times larger than Wave snails at maturity. Our finding of a QTL for size, a classic example of a highly polygenic trait, makes *L. saxatilis* rather exceptional, and might have facilitated evolution of differently sized ecotypes (Reid 1996; Johannesson et al. 2010). Influence of inversions on adult size has also been described in *Drosophila* (Kapun and Flatt 2019) and seaweed flies (Butlin et al. 1982) and might be due to the combined effect of multiple small effect loci within inversions.

LG 17 showed clear QTL peaks for several parameters describing shell shape and aperture size and position. Shape is under divergent selection in the two different habitats. Under wave action globular shells as well as a larger foot area help snails to remain attached to the rock surface and decrease the risk of dislodgment (Le Pennec et al. 2017). In contrast, under crab predation, narrower apertures protect snails from being pulled out and high‐spired shells allow them to retract further inside the shell (Johannesson 1986; Boulding et al. 2017).

Interestingly, an inverted region on LG 14 that exhibited a high number of non‐neutral SNPs in cline analyses (Westram et al. 2018) showed some influence on weight but not on other traits studied despite segregation of the inversion in the F2. We should keep in mind that adaptation to the different habitats may include more traits than those measured here, and may involve, for instance, important physiological traits (Sokolova and Pörtner 2003; Panova and Johannesson 2004).

Color categories (black and beige) had been shown to vary clinally across the contact zone at the Swedish site. SNPs associated with these colors were found on LG 5 and LG 9 (Westram et al. 2018). In contrast, here we found a clear association with LG 6 and LG 17 for color traits. The way we analyzed color as a continuous variable (rgb‐value) might explain this discrepancy. However, variation in color was not high among F2 individuals, which might have limited our precision for estimating relevant effects. Consequently, the high estimates obtained for color in variance partitioning (Supporting information Table S5, Fig. 4) should be interpreted with caution.

### INSIGHTS INTO GENETIC ARCHITECTURE OF LOCAL ADAPTATION BY USING COMPLEMENTARY APPROACHES

A classical question is whether adaptation is mainly due to some large effect loci or mainly polygenic. Polygenic architecture may be common and often QTL or association studies fail to detect significant loci for heritable traits if each individual locus has only a small effect (“missing heritability”) (Pritchard and Di Rienzo 2010; Rockman 2012). In our case, we had the a priori expectation that inversion regions previously identified as enriched for genetic differentiation should have a strong influence on phenotypic divergence. A QTL scan was thus a useful approach and indeed confirmed our expectation for some traits. Most significant peaks in our QTL analysis mapped to inverted regions on LG 6 and 17 that were strong candidates for local adaptation in previous studies. However, inversions, large blocks with little to no recombination, may lead to a detection bias toward these regions (Noor et al. 2001; Roesti 2018). Even without a clustering of important loci in these regions, statistical power for any association analysis between genetic markers and phenotypes is increased. Combining QTL mapping with variance partitioning across LGs might help in two ways. First, it can circumvent this detection bias by showing that candidate chromosomes with inversions explain high amounts of phenotypic variance. In addition, it gives a more nuanced overview than focusing on inversion regions only. It can help to identify genomic regions containing many loci of effects that are too small to be detected individually (Riggio et al. 2013). This higher sensitivity resulted in significant results for many traits without significant peaks in the QTL analysis. We can thus give a more comprehensive picture, which also allows a better evaluation of the importance of inversions in relation to the remaining genomic background. Testing effects of inversion genotypes directly mainly confirmed results of the QTL analysis and variance partitioning and provided additional support for the effects of the different arrangements on phenotypes (Supporting information Fig. S5, Table S7). Interestingly, some inversions showed significant effects although no QTL in these regions was detected. This could indicate that, in these cases, position effects potentially influencing gene expression are more important than allelic content. Alternatively, testing for genotypes directly may integrate effects of all loci within the inversion region and may, thus, increase the statistical power similar to variance partitioning.

Almost all traits show clear deviations from the pattern expected under a purely polygenic architecture, where variance explained should increase with chromosomal length (Fig. 4A). This indicates presence of large effect loci or a nonuniform distribution of loci and clustering in certain regions. In line with our expectation and QTL analysis (see Figs. 2, 3), LG 6 and LG 17 that harbor inversions involved in genetic ecotype differentiation were clearly identified as outliers in variance partitioning for several traits (Fig. 4). However, some discrepancies exist for LG 5 and 12, which neither showed significant peaks in the QTL analysis, but both clearly stood out in variance partitioning. This may suggest that contribution of these LGs to phenotypic variance is due to a clustering of many loci of small effects that cannot be detected individually by QTL analysis but only by variance partitioning that integrates the effect of the whole LG.

Particularly interesting is LG 12 that most likely includes a sex‐determination locus. *Littorina saxatilis* does not seem to have heteromorphic sex chromosomes (García‐Souto et al. 2018) and the exact sex determining mechanism is unknown. In other systems, inversions are involved in the evolution of sex chromosomes (Rice 1987; Lenormand 2003; Connallon et al. 2018) as they can suppress recombination and maintain sets of alleles under sexually antagonistic selection. Coupling of alleles with sex‐specific benefits to the sex determining locus can ultimately lead to the evolution of sex chromosomes. Some of the traits associated with LG 12 (Height and Width Growth and Aperture Size) showed differences between sexes (Larsson et al. 2020). However, it is unknown whether and how they influence fitness in males and females.

RHM, where each linkage group is divided into equally sized smaller regions was used to get more information at a finer scale. In the case of Height and Width Growth, we could show in this way that high variance explained by LG 2 is not solely caused by its length and a simple cumulative effect of many loci evenly distributed along the LG. RHM showed an accumulation of regions contributing disproportionally to phenotypic variance in the center of this LG consistent with an enrichment of Crab/Wave outliers that was found before (Morales et al. 2019). Other mechanisms than inversions can lead to high LD and clusters of loci contributing to divergence (Rafajlović et al. 2016; Burri 2017; Roesti 2018). Low recombination and clustering of adaptive loci may be under positive selection in situations of divergent selection with gene flow. Accumulation of differentiated loci close to the centromere during speciation with gene flow had also been described (Carneiro et al. 2009) and might be an explanation for the clustering of candidate regions in the center of LG 2. However, in our experiment the ability to reliably detect clusters of adaptive loci is limited by strong LD between regions on the same LG. Since, we worked with a F2‐cross there had not been much opportunity for recombination and adjacent regions show often the same estimate.

### GENETIC CORRELATIONS FACILITATED ECOTYPE EVOLUTION AND CONTRIBUTED TO ADAPTATION

Using bivariate animal models for estimation of genetic correlation provided insights into the extent to which different traits share a genetic basis and may, thus, be prevented from evolving independently. Genetic correlations among traits may either increase or decrease the rate of adaptation, depending on the direction of maximum genetic variance relative to selection acting on the different traits (Lande and Arnold 1983; Hansen and Houle 2008; Stinchcombe et al. 2014). They can prevent adaptation if a correlated trait evolves in a direction that disfavors adaptation or they can increase and facilitate evolution if multivariate selection is in line with genetic covariances. We could show that features that characterize ecotypes in the field are genetically correlated in a way that facilitates adaptation. For example, thicker shells, elongate shape and narrower apertures are features that are genetically correlated and are all under positive selection in the Crab habitat. This may explain the success of *L. saxatilis* in rapidly evolving locally adapted populations multiple times (Johannesson et al. 2010; Butlin et al. 2014; Ravinet et al. 2016).

Genetic correlations alone do not provide any information on whether they are caused by pleiotropic effects, strong linkage between loci or which regions in the genome contribute to them. Here, the QTL analysis gave additional insights by showing that some inversions influence several traits (Fig. 4B). If adaptation depends on alleles at several loci, reduced recombination between them will be positively selected under gene flow. An inversion containing several loci can thus serve as a toolkit for adaptation to different habitats and both facilitate and accelerate formation of locally adapted ecotypes if the alleles combined inside an inversion are in line with the selection pressures associated with a certain habitat. Inversion polymorphisms in an ancestral population, potentially maintained by balancing selection (Faria et al. 2019b), could, thus, lead to a rapid and repeated formation of ecotypes as was found in sticklebacks (Roesti et al. 2015) and saltmarsh beetles (Van Belleghem et al. 2018).

Although this hypothesis of beneficial recombination suppression has been very popular and is in line with the frequent observation of inversions involved in ecotype formation and speciation (Kirkpatrick and Barton 2006; Ortiz‐Barrientos et al. 2016; Charlesworth and Barton 2018; Wellenreuther et al. 2019), empirical evidence remains elusive given the complexity of detecting at least two adaptive loci inside an inversion (but see for example Fuller et al. 2017; Lee et al. 2017; Coughlan and Willis 2019). Here, we argue that our observation of some inversions explaining variation in more than one trait is suggestive for adaptive recombination suppression. However, without knowing the exact genetic basis and identification of responsible genes we cannot confirm that this is caused by multiple loci inside the inversion. Specific mechanisms by which inversions can influence phenotypes are diverse. They can have a strong and direct influence on phenotypes when genes at breakpoints are disrupted. Independent of allelic contents, directionality of an inversion can influence phenotypes by rearranging regulatory regions and changing gene expression (Lavington and Kern 2017; Huang et al. 2018; Said et al. 2018). It is, thus, also possible that observed associations with several traits are caused by pleiotropic effects.

Genetic covariances can also help us to disentangle the causative drivers of phenotypic clines observed in nature. Two potential problems when analyzing clines are high confounding of environmental factors and also identifying the target of selection when many traits change simultaneously. It is possible that some of the phenotypic clines observed in *L. saxatilis* could be the result of indirect selection acting on other traits. We found evidence that color was influenced by an inversion on LG 6 that contribute to many other traits under divergent selection. Thus, coloration might be cosegregating with other traits directly targeted by selection, potentially explaining the large amount of color polymorphism in this species (Johannesson and Butlin 2017).

The exact mechanisms by which inversions influence phenotypes are still unknown. Measuring gene expression, testing whether transcript abundance for reads mapped to inversion regions differs between karyotypes, may help to test whether allelic content or directionality are more important for phenotypic variation. While tight linkage between alleles within an inversion might have facilitated adaptation to Crab/Wave habitats, it also means a reduced evolutionary flexibility since some traits cannot evolve independently. For instance, a high genetic correlation between shape and aperture size found here indicates that evolution of forms with narrow apertures but the globular wave shape might be unlikely or limited. In contrast, correlation between size measures and shape is lower meaning they can evolve independently. *Littorina saxatilis* shows a large range of differently adapted ecotypes with different shapes and sizes (Reid 1996; Johannesson et al. 2010). It remains an open question how widespread specific inversions are, and whether the same ancestral inversion polymorphism was repeatedly involved in ecotype development.

## AUTHOR CONTRIBUTION

RKB and KJ conceived the study; KJ, AMW, RKB, JL, ARL, and EML collected data; ELK, HM, JL, and RF analyzed data; ELK drafted the initial version of the manuscript, and all authors contributed to later versions of the manuscript.

## CONFLICT OF INTEREST

The authors declare no conflict of interest.

## DATA ARCHIVING

Data and scripts are available from the Dryad Digital Repository. The dyrad doi is https://doi.org/10.5061/dryad.zgmsbccb4.

## Supporting information

Figure S1: Linkage Map
Figure S2: QTL scans for relative thickness and boldness
Figure S3: Regional heritability for color, shell length, thickness, Aperture Shape, Width Growth, Aperture Position, Aperture Size, relative thickness
Figure S4: Phenotypic correlations between traits
Figure S5: Inversion effects on phenotypes
Table S1: Map position of putatively inverted regions
Table S2: Inversion genotypes of F1 and F2 individuals
Table S3: Correspondence between linkage groups of the new map with previous map
Table S4: Results of QTL analysis
Table S5: Results of variance partitioning across linkage groups
Table S6: Genetic covariances
Table S7: Inversion effects on phenotypes: statistical test results
Table S8: List of all capture sequencing probes
Appendix S1: Crossing experiment, genotyping, and validation of full‐sib families

Supplementary Material

Supplementary Material
