# Supplementary material for: Genetic variation for adaptive traits is associated with polymorphic inversions in Littorina saxatilis
Source: Evol Lett. 2021 May 7;5(3):196–213. doi: 10.1002/evl3.227 (PMC8190449; doi:10.1002/evl3.227)
Supplement: Supplementary file 1 — Figure S1: Linkage Map Figure S2: QTL scans for relative thickness and boldness Figure S3: Regional heritability for color, shell length, thickness, Aperture Shape, Width Growth, Aperture Position, Aperture Size, relative thickness Figure S4: Phenotypic correlations between traits Figure S5: Inversion effects on phenotypes Table S1: Map position of putatively inverted regions Table S2: Inversion genotypes of F1 and F2 individuals Table S3: Correspondence between linkage groups of the new map with previous map Table S4: Results of QTL analysis Table S5: Results of variance partitioning across linkage groups Table S6: Genetic covariances Table S7: Inversion effects on phenotypes: statistical test results Table S8: List of all capture sequencing probes Appendix S1: Crossing experiment, genotyping, and validation of full‐sib families [file EVL3-5-196-s001.pdf]

Supporting Information

Genetic variation for adaptive traits is associated with polymorphic inversions in *Littorina saxatilis*

E. L. Koch[1], H. E. Morales[2,7], J. Larsson[1], A. M. Westram[1,3], R. Faria[1,4], A. R. Lemmon[5], E. M. Lemmon[6], K. Johannesson[7] *, R. K. Butlin[1,7]*

1 Department of Animal and Plant Sciences, University of Sheffield, Sheffield, UK
2 Evolutionary Genetics Section, Globe Institute, University of Copenhagen, Copenhagen, Denmark
3 IST Austria, Klosterneuburg, Austria
4 CIBIO-InBIO, Centro de Investigação em Biodiversidade e Recursos Genéticos, Universidade do Porto, Vairão, Portugal
5 Department of Scientific Computing, Florida State University, Tallahassee, Florida
6 Department of Biological Science, Florida State University, Tallahassee, Florida
7 Department of Marine Sciences, University of Gothenburg, Strömstad, Sweden

* These authors contributed equally to this work.

Correspondence: e.koch@sheffield.ac.uk

Contents

Figure S1: Linkage Map
Figure S2: QTL scans for relative thickness and boldness
Figure S3: Regional heritability for colour, shell length, thickness, Aperture Shape, Width Growth, Aperture Position, Aperture Size, relative thickness
Figure S4: Phenotypic correlations between traits
Figure S5: Inversion effects on phenotypes

Table S1 : Map position of putatively inverted regions
Table S2 : Inversion genotypes of F1 and F2 individuals
Table S3 : Correspondence between linkage groups of the new map with previous map
Table S4: Results of QTL analysis
Table S5: Results of variance partitioning across linkage groups
Table S6: Genetic covariances
Table S7: Inversion effects on phenotypes: statistical test results
Table S8: List of all capture sequencing probes

Appendix S1: Crossing experiment, genotyping, and validation of full-sib families

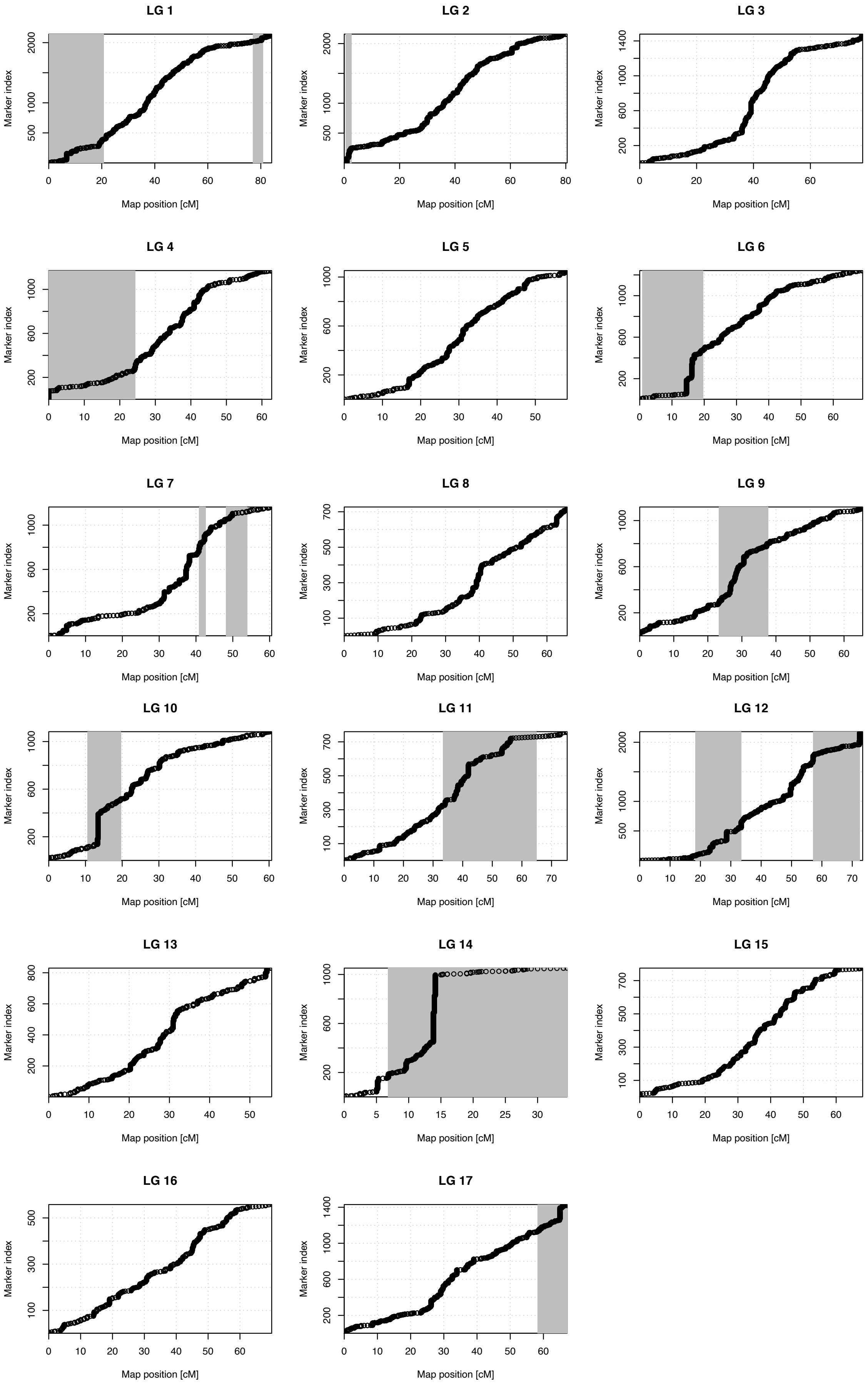

Figure S1: Mapping positions of markers for each linkage group (LG) with position on the x-axis and marker index on the y-axis. Grey background indicates the regions of putative inversion based on Faria et al. 2019, Table 1 (doi:10.1111/mec.14972). The positions in our map are based on markers that are in common with the previous linkage map. The exact positions of the inverted regions can thus only be approximated since markers at the utmost boundaries of the inversions were not always present in our data set (see Table S1). Regions of reduced recombination are visible by many markers that share the same map position and are often within the inverted regions.

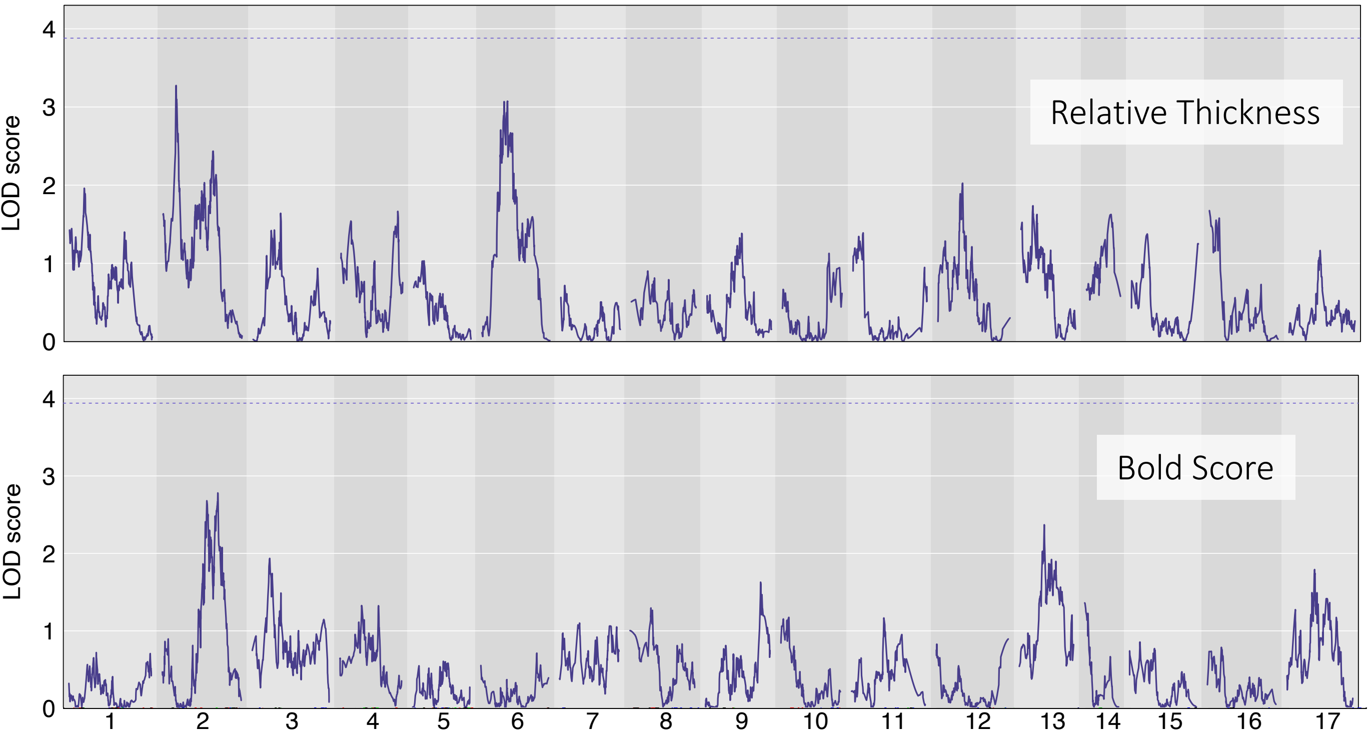

Figure S2: QTL scans for relative shell thickness (size independent) and Bold Score (time until emergence after disturbance). Dashed lines indicate genome-wide significant thresholds (P = 0.05)

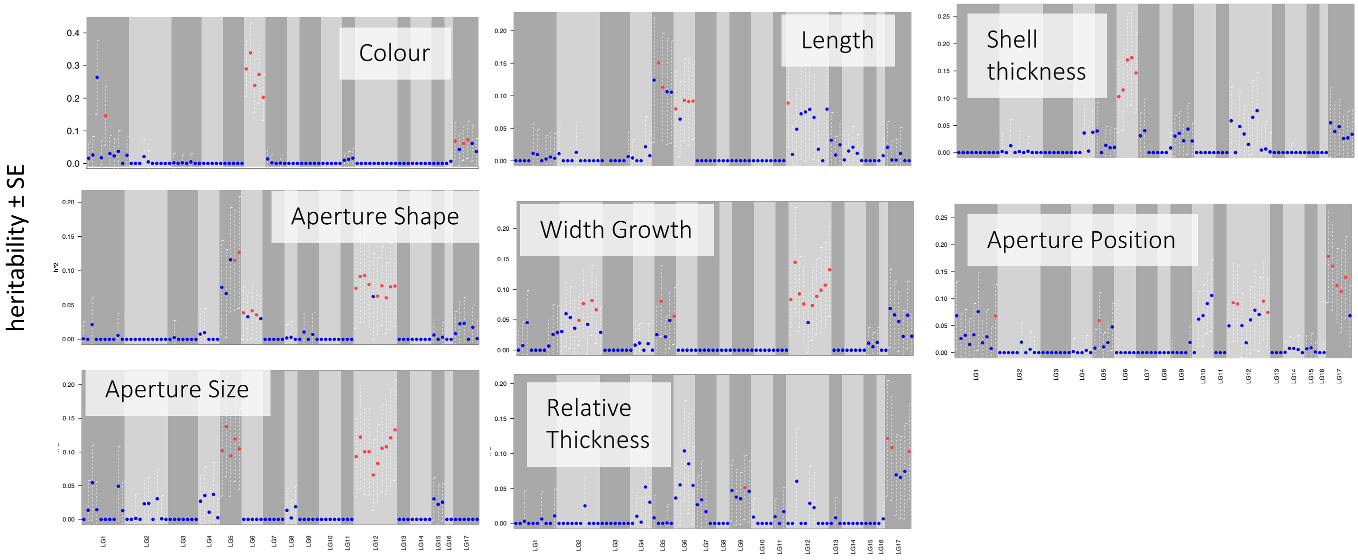

Figure S3: Regional heritability for colour, shell length, thickness, Aperture Shape, Width Growth, Aperture Position, Aperture Size, relative thickness. Each region consisted of 200 adjacent markers. Significant estimates are shown in red.

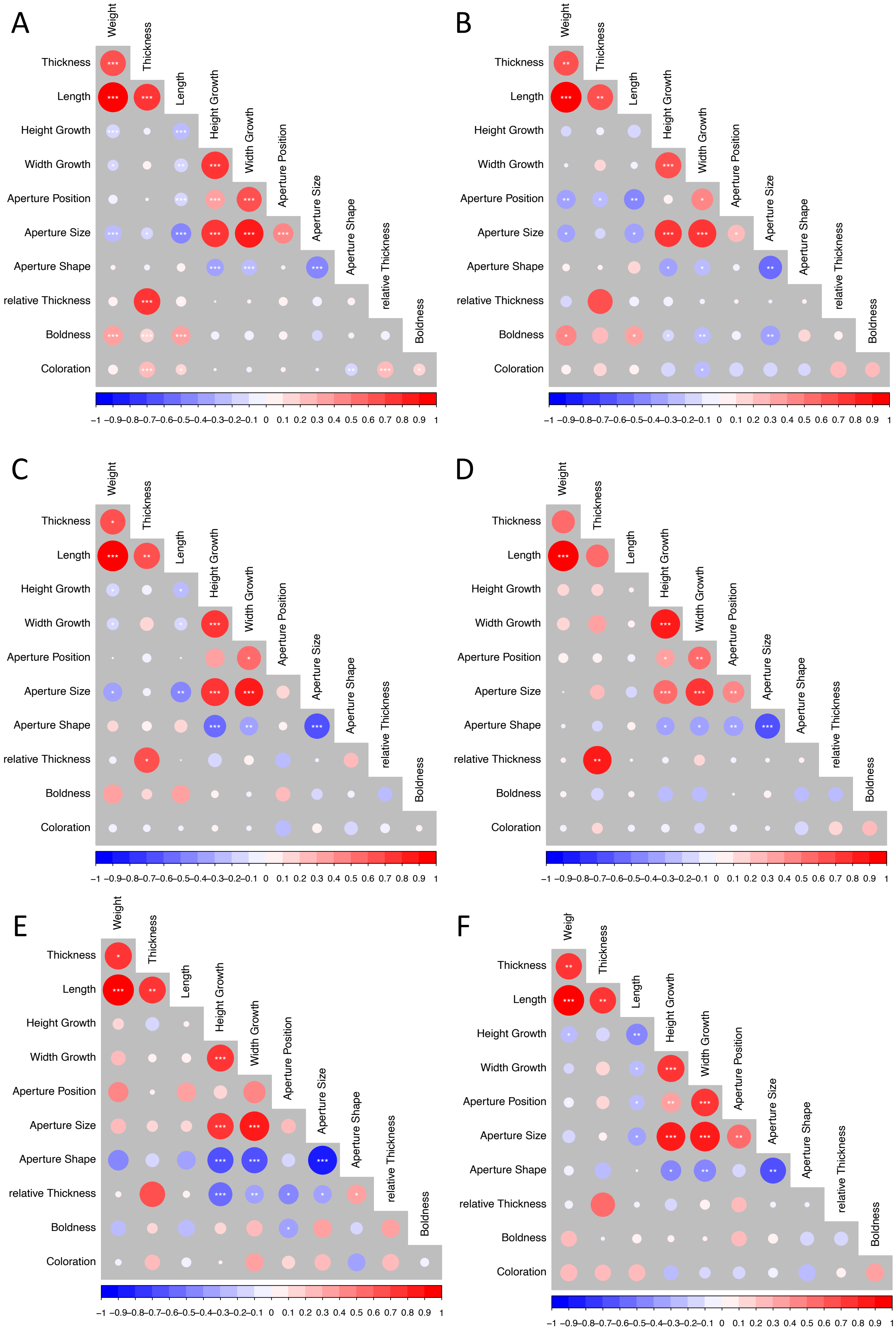
A
Weight
Thickness
Length
Height Growth
Width Growth
Aperture Position
Aperture Size
Aperture Shape
relative Thickness
Boldness
Coloration
−1 −0.9 −0.8 −0.7 −0.6 −0.5 −0.4 −0.3 −0.2 −0.1 0 0.1 0.2 0.3 0.4 0.5 0.6 0.7 0.8 0.9 1
B
Weight
Thickness
Length
Height Growth
Width Growth
Aperture Position
Aperture Size
Aperture Shape
relative Thickness
Boldness
Coloration
−1 −0.9 −0.8 −0.7 −0.6 −0.5 −0.4 −0.3 −0.2 −0.1 0 0.1 0.2 0.3 0.4 0.5 0.6 0.7 0.8 0.9 1
C
Weight
Thickness
Length
Height Growth
Width Growth
Aperture Position
Aperture Size
Aperture Shape
relative Thickness
Boldness
Coloration
−1 −0.9 −0.8 −0.7 −0.6 −0.5 −0.4 −0.3 −0.2 −0.1 0 0.1 0.2 0.3 0.4 0.5 0.6 0.7 0.8 0.9 1
D
Weight
Thickness
Length
Height Growth
Width Growth
Aperture Position
Aperture Size
Aperture Shape
relative Thickness
Boldness
Coloration
−1 −0.9 −0.8 −0.7 −0.6 −0.5 −0.4 −0.3 −0.2 −0.1 0 0.1 0.2 0.3 0.4 0.5 0.6 0.7 0.8 0.9 1
E
Weight
Thickness
Length
Height Growth
Width Growth
Aperture Position
Aperture Size
Aperture Shape
relative Thickness
Boldness
Coloration
−1 −0.9 −0.8 −0.7 −0.6 −0.5 −0.4 −0.3 −0.2 −0.1 0 0.1 0.2 0.3 0.4 0.5 0.6 0.7 0.8 0.9 1
F
Weigt
Thickness
Length
Height Growth
Width Growth
Aperture Position
Aperture Size
Aperture Shape
relative Thickness
Boldness
Coloration
−1 −0.9 −0.8 −0.7 −0.6 −0.5 −0.4 −0.3 −0.2 −0.1 0 0.1 0.2 0.3 0.4 0.5 0.6 0.7 0.8 0.9 1

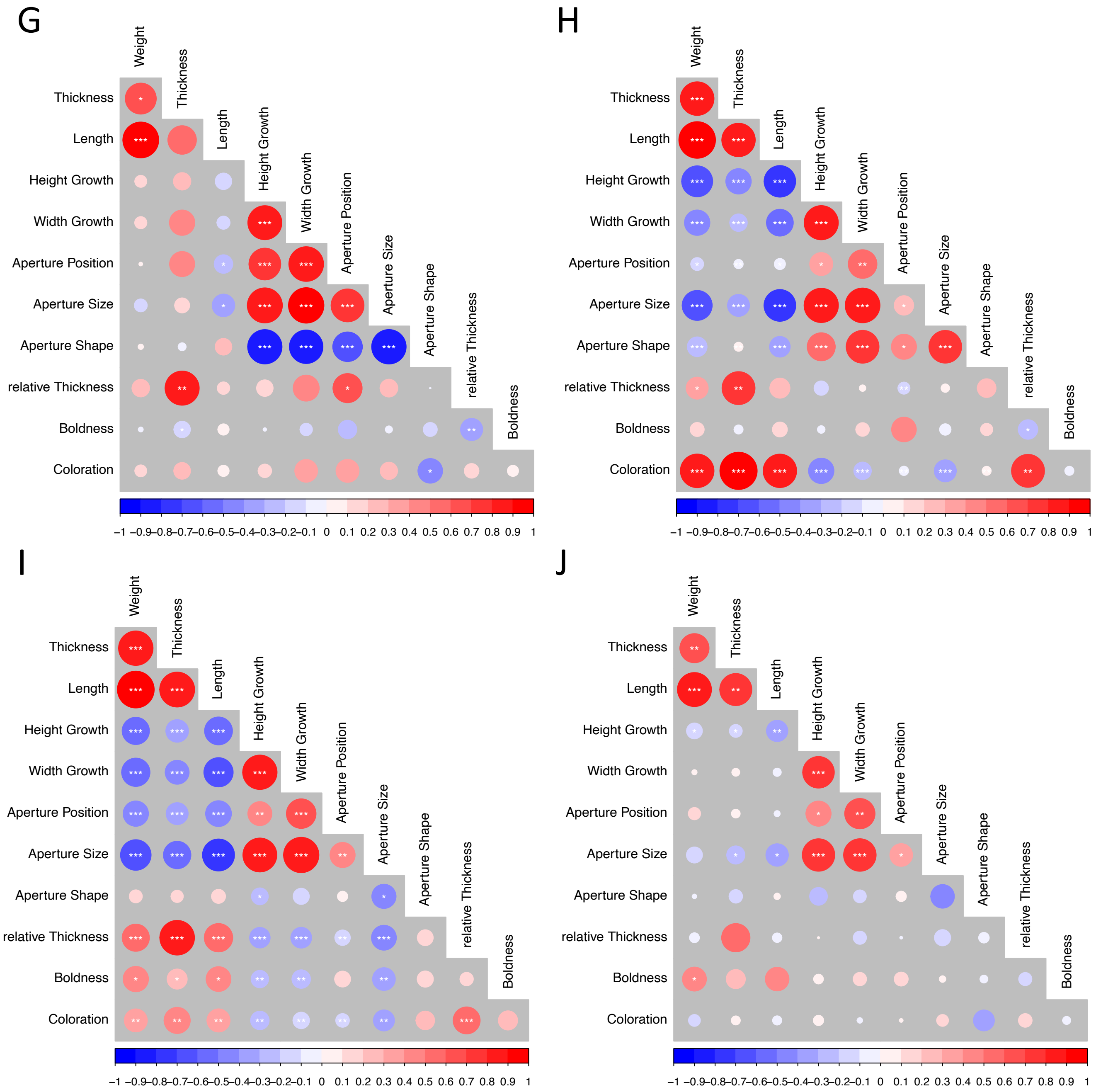

Figure S4: Phenotypic correlations between traits for all individuals **(A)** and separately for each family **(B-J)**. Circles are proportional to correlation coefficients. Significance: *P < 0.05; **P < 0.01; ***P<0.001. **A**: all F2 individuals; **B**: family81.1; **C**: family82.1, **D**: family83.1; **E**: family83.2; **F**: family91.1; **G**: family91.2; **H**: family92.1; **I**: family92.2; **J**: family93. Information for the different families can be found in the appendix.

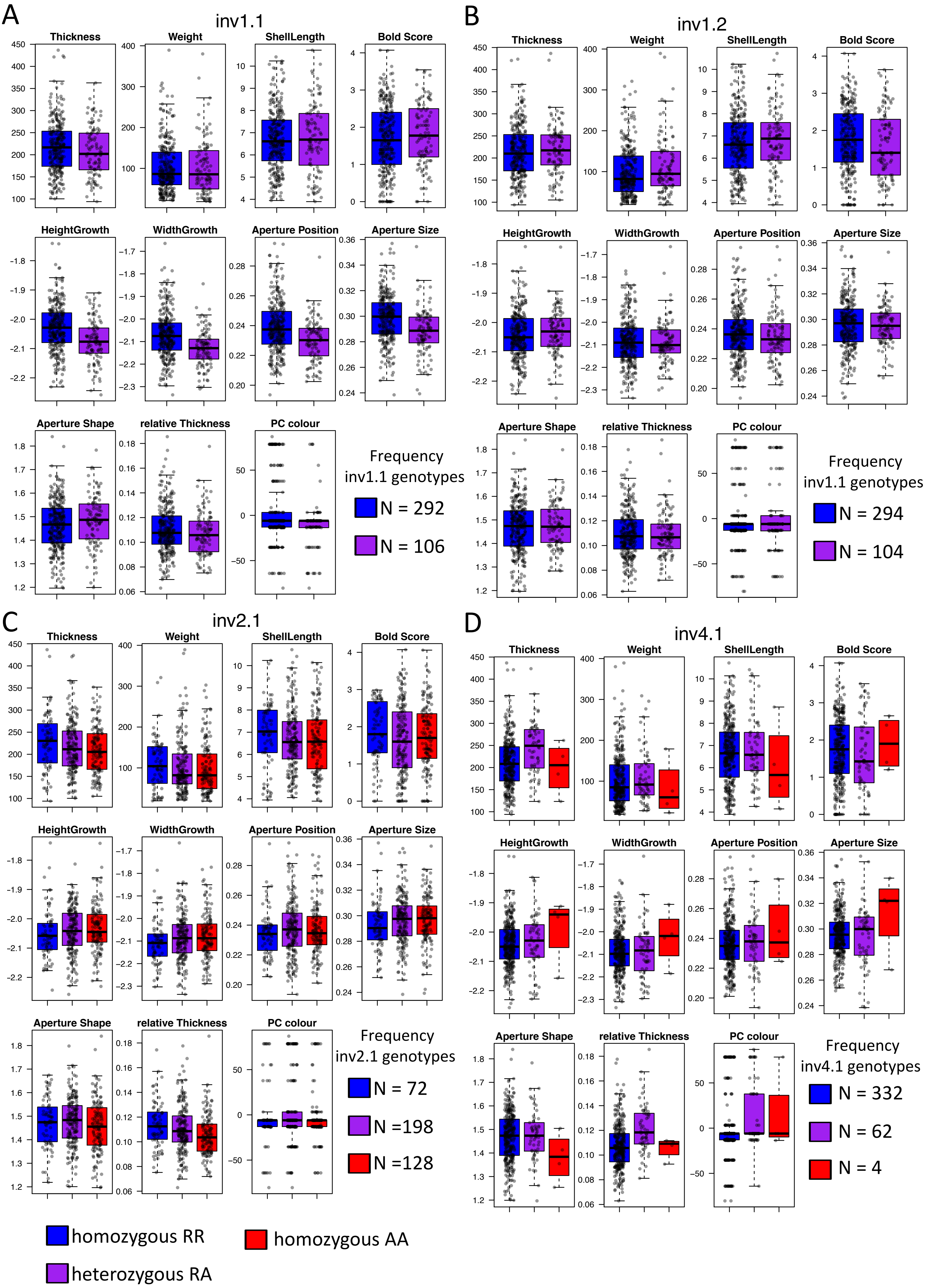
A
inv1.1
B
inv1.2
C
inv2.1
D
inv4.1
Thickness
Weight
ShellLength
Bold Score
HeightGrowth
WidthGrowth
Aperture Position
Aperture Size
Aperture Shape
relative Thickness
PC colour
Frequency inv1.1 genotypes
N = 292
N = 106
Frequency inv1.1 genotypes
N = 294
N = 104
Frequency inv2.1 genotypes
N = 72
N =198
N =128
Frequency inv4.1 genotypes
N = 332
N = 62
N = 4
homozygous RR
heterozygous RA
homozygous AA

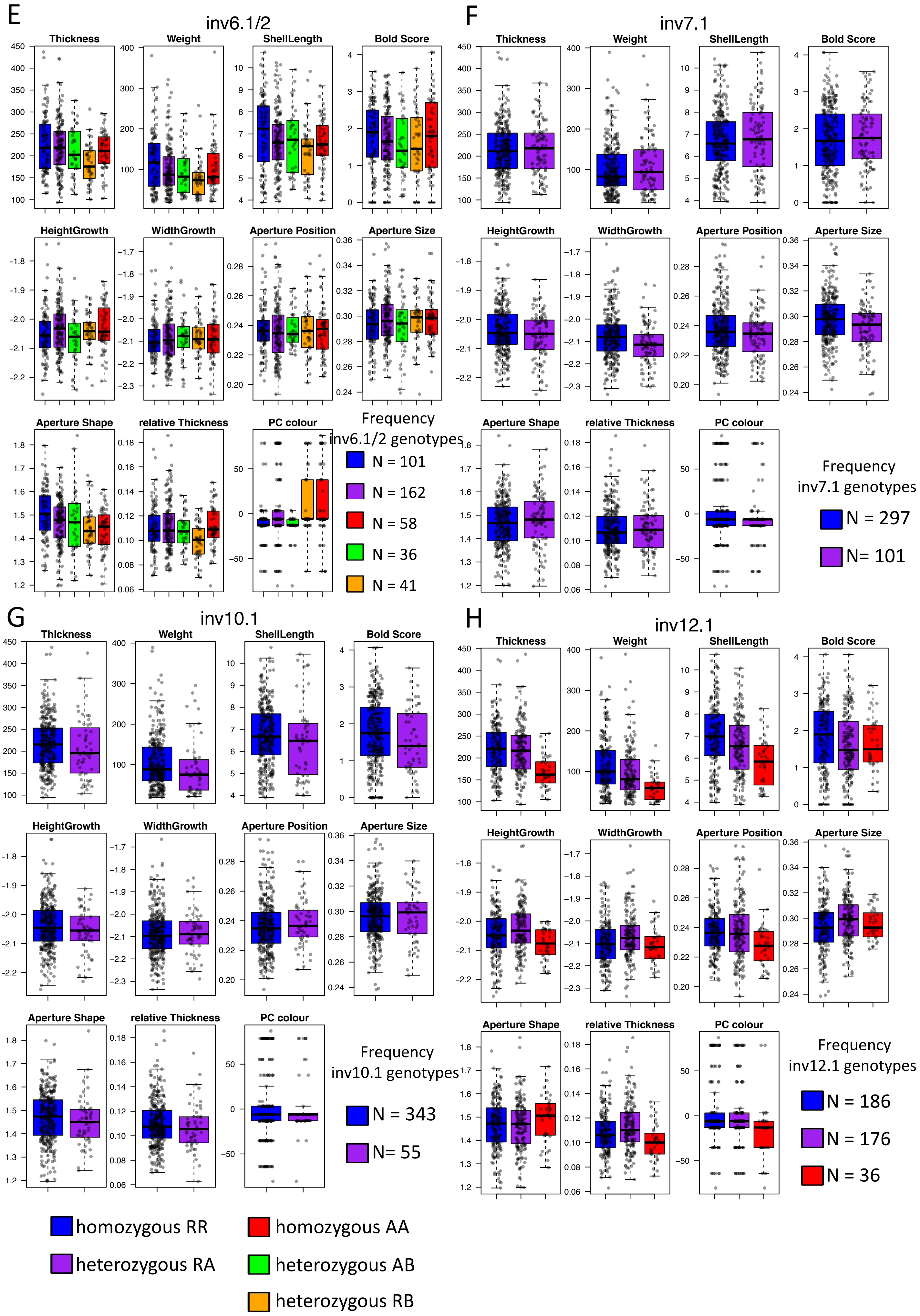
E
inv6.1/2
Thickness
Weight
ShellLength
Bold Score
HeightGrowth
WidthGrowth
Aperture Position
Aperture Size
Aperture Shape
relative Thickness
PC colour
Frequency
inv6.1/2 genotypes
N = 101
N = 162
N = 58
N = 36
N = 41
F
inv7.1
Thickness
Weight
ShellLength
Bold Score
HeightGrowth
WidthGrowth
Aperture Position
Aperture Size
Aperture Shape
relative Thickness
PC colour
Frequency
inv7.1 genotypes
N = 297
N= 101
G
inv10.1
Thickness
Weight
ShellLength
Bold Score
HeightGrowth
WidthGrowth
Aperture Position
Aperture Size
Aperture Shape
relative Thickness
PC colour
Frequency
inv10.1 genotypes
N = 343
N= 55
H
inv12.1
Thickness
Weight
ShellLength
Bold Score
HeightGrowth
WidthGrowth
Aperture Position
Aperture Size
Aperture Shape
relative Thickness
PC colour
Frequency
inv12.1 genotypes
N = 186
N = 176
N = 36
homozygous RR
homozygous AA
heterozygous RA
heterozygous AB
heterozygous RB

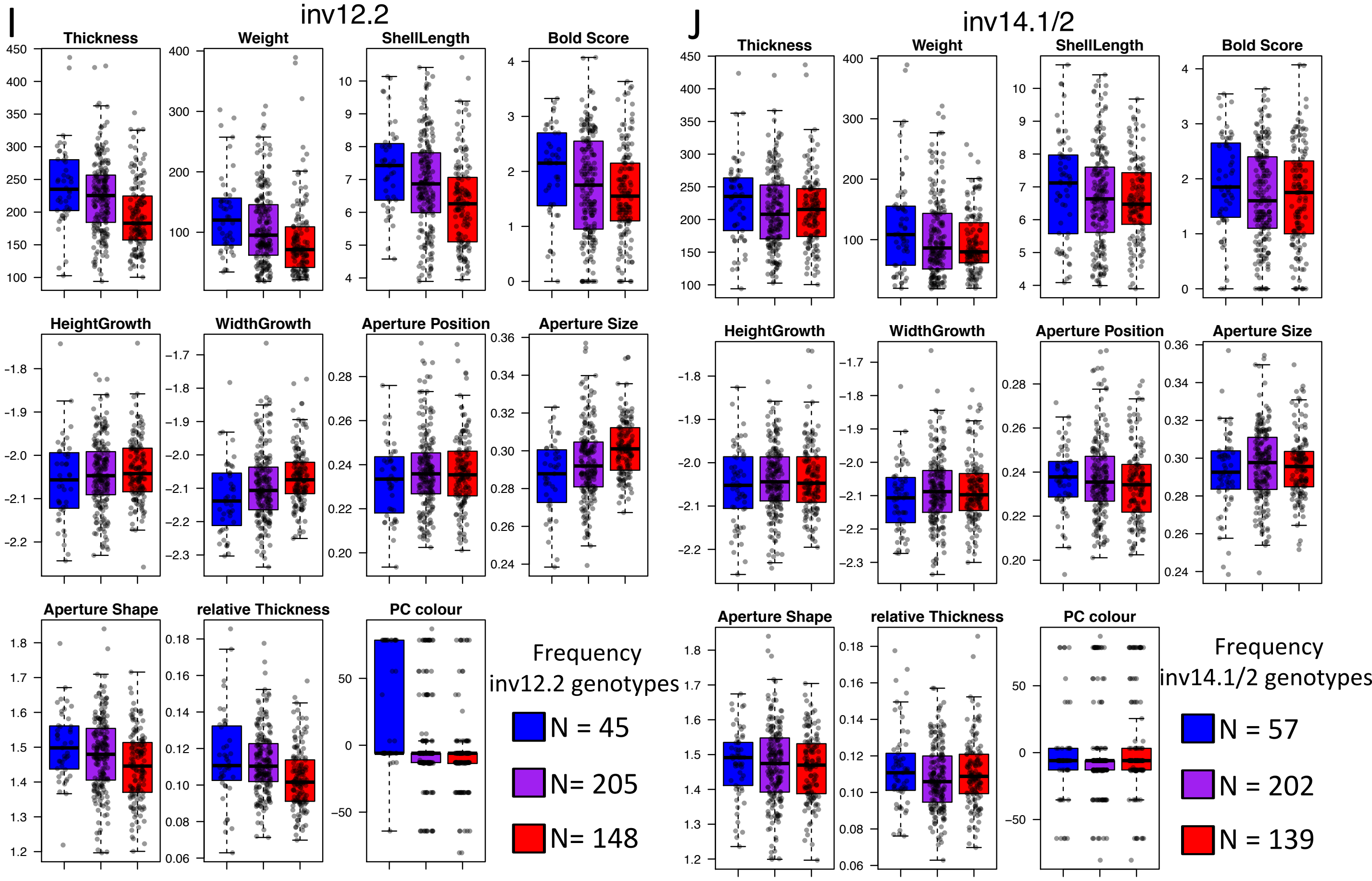

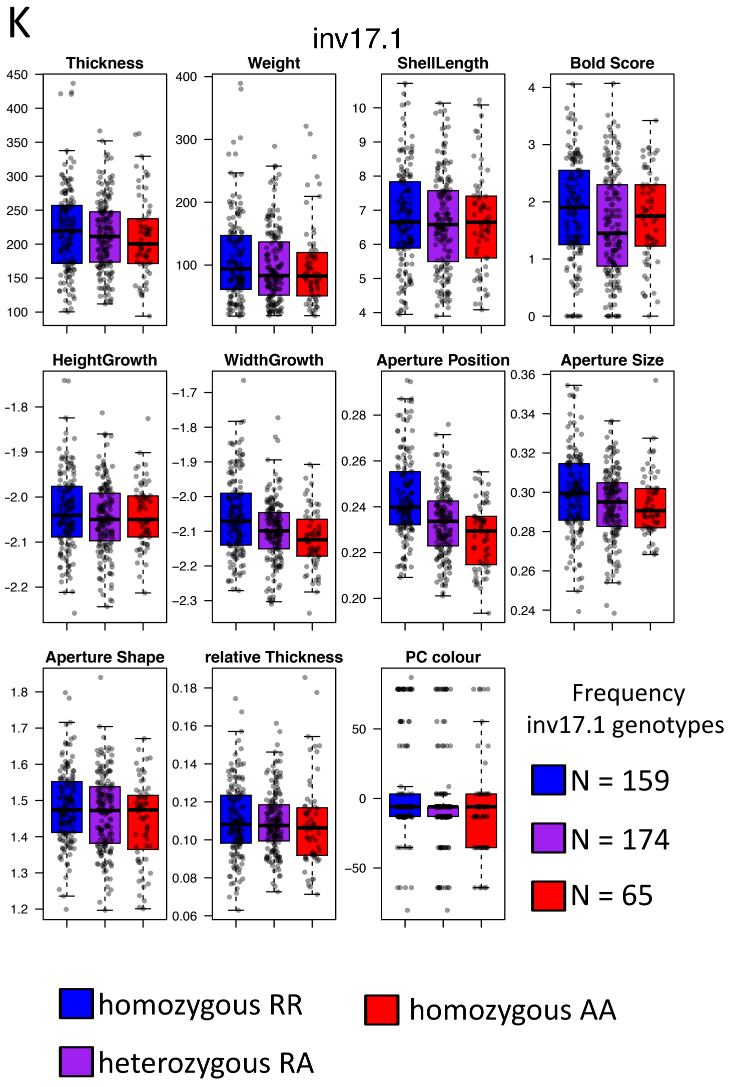

Figure S5: Inversion effects on phenotypes: Thickness [µm] (thickness of shell); Weight [mg]; Shell length [mm], Bold Score (log time until crawling out after disturbance); Height Growth, Width Growth, Aperture Position, Aperture Size , Aperture Shape: see explanations Figure 1 of the main manuscript; relative Thickness (size corrected shell thickness); Colour: PC1 of rgb colour values. Individuals were genotyped using a PCA based on SNPs within inverted regions. Arrangement R is more common in the Crab ecotype except for inversion 12.1 where the frequency of both arrangements is very similar. For statistical testing of inversion effects see Table S7 in Supporting Information
