# Supplementary material for: Genetic variation for adaptive traits is associated with polymorphic inversions in Littorina saxatilis
Source: Evol Lett. 2021 May 7;5(3):196–213. doi: 10.1002/evl3.227 (PMC8190449; doi:10.1002/evl3.227)
Supplement: Supplementary file 3 — Supplementary Material [file EVL3-5-196-s003.pdf]

Appendix S1

Crossing and confirmation of relationships between individuals

Crossing was performed between Crab and Wave ecotype individuals collected on the Swedish West Coast at Ängklåvebukten (58.8697°, 11.1197°), where both ecotypes occur in close proximity (see also Westram et al. 2018). The parental female snails were brought into the lab as juveniles and raised in isolation until maturity to prevent uncontrolled matings. The parental males were brought in as adults. Two virgin Crab-females were crossed with two Wave-males resulting in two F1-families (Figure I). Three males and three females of each F1-family were then crossed reciprocally with an individual from the other family to produce six F2-families. F1 and F2 generations were raised in large tanks provided with flow-through sea water from 40 m depth at ambient temperature (5-15 degrees depending on season) and salinity (approx. 30 psu) filtered through sand-filters. Snails grazed the biofilm of microalgae growing inside the tanks under the illumination of daylight lamps. Generation time in the lab was around 6-12 months depending on water temperature.

We performed targeted re-sequencing as described in Faria et al. (2019) and Westram et al. (2018), using a total of 25,000 (120 bp) enrichment probes (Supporting Information Table S8), 20,000 that were informative in previous experiments and 5000 novel probe regions chosen from random contigs of the *Littorina saxatilis* genome assembly that were not placed on the existing genetic map, with the intention of extend the linkage map coverage. Raw reads were processed as described in Faria et al (2019). In short, the pipeline consisted in read-trimming with Trimmomatic v. 0.36 (Bolger et al. 2014), quality control with FastQC v0.11.5 (Andrews 2010), read-mapping with BWA v0.7.15 (Li and Durbin 2009), PCR duplicate removal and InDel re-alignment with Picard v. 1.138 (http://broadinstitute.github.io/picard/) and SNP calling with GATK UnifiedGenotyper v3.7-0 (Depristo et al. 2011). We aggressively filtered the resulting genotypes to retain a set of high confidence SNPs with vcftools (Danecek et al. 2011) and vcfilter from vcflib (https://github.com/vcflib/vcflib). The final set of SNPs consisted of 22,759 sites with an average depth of 44X (SD=22X) across individuals, a minimum individual depth of 8X, a minimum base and mapping qualities of 40, a maximum missingness per individual of 0.5% (average = 0.14%), a maximum missingness per site of 17% (average = 0.12%) and a minor allele frequency higher than 5%.

Inversion genotypes of F1 parents and F2 offspring (after confirming their relationships, see below) were inferred using the genotypes (extracted from vcf -file using vcftools) of the same marker data that were used for linkage map construction. SNPs with unknown map position and more than 1000 bp away from mapped SNPs in the previous linkage map were removed, as in Westram et al. (2018). The original map position of the remaining SNPs was then replaced by their position in our map. A principal component analyses (PCA) using the SNPs located within each putatively inverted region (with boundaries defined according to our map) was implemented with the R package PCADAPT (Luu et al. 2017) including all individuals,

as in Faria et al. (2019). Groups of individuals across the PC1 (without intermediates) were identified using the R function "KMEANS" and confirmed/adjusted visually. For each inversion, the number of groups observed for each F1 couple and their offspring varied between one, two or three, representing a cross between two homozygotes for one arrangement, a cross between a homozygote for one arrangement and a heterozygote, and a cross between two inversion heterozygotes, respectively. The only exception was observed for the complex inversion LGC6.1/2 where six groups of genotypes were observed, as expected for the presence of three possible arrangements in the analysed individuals. The inversion genotyping consistency was subsequently confirmed by comparing the genotypes of offspring and their (known) parents. The most likely inversion genotype of each unknown parent was inferred based on the distribution of its offspring and mate genotypes. A second PCA was subsequently implemented, where genotypes from the two crab parents of the crab linkage map and 28 individuals from the crab end of the transect of the same source population obtained from Westram et al. (2018) and Faria et al. (2019) were also included. The goal of this second PCA was to identify the most common arrangement in the crab ecotype (R) and to genotype the F1 parents and F2 offspring individuals as RR, RA or AA (and RB, AB, and BB for LGC6.1/2), where A (and B) are alternative arrangements. The only exception was observed for LGC12.2, where R and A are equally frequent in the crab ecotype, and each arrangement was classified as R or A in a random manner.

Confirmation that individuals of each presumed family are true full sibs is crucial for linkage map construction. False classification of more distantly related individuals as full-sibs would inflate the map since the number of recombination events would have been much higher than assumed.

We calculated genomic relationships among presumed F2 individuals following VanRaden (2008), as implemented in the Rpackage "AGHmatrix" (Amadeu et al. 2016). We also tested relationships using the IBD module in LepMap 3 (Rastas 2017). To avoid using genotypes that were curated by our input pedigree (this module takes the output of the "ParentCall2" module in LepMap 3 and not vcf-files directly) we set raw=1 in "ParentCall2" to prevent LepMap using pedigree information while producing proper input format for the IBD module. The results were consistent with the genomic relationships obtained from the method by VanRaden (2008).

We tested the relationship of all individuals of a presumed family with their presumed parents and to each other. For each family we expected that individuals were either full-sibs, in line with the original crossing scheme, half-sibs, potentially caused by using non-virgin females that stored sperm of previous matings, or unrelated, presumably due to contamination from other tanks. Identifying individuals that share the same father and mother is the most reliable way to identify full-sibs. Unfortunately, genetic data for F1 parents were not always available (Table I) since these individuals died before DNA collection or the true parent was not

sequenced. In these cases, we could only test relationship with one potential parent or with other F2 individuals.

In all cases, the presumed families consisted of several genetic sub-groups (Figure II-VII) indicating that not all of them were true full-sibs. For some of the identified sub-groups we found that both presumed parents were wrong and true parents could not be identified. Sometimes they showed intermediate relationships with the intended parents suggesting that they were offspring of other F1-individuals (avuncular relationship to planned parent). We assumed the identified subgroups to be full-sibs if they formed a cluster based on their genomic relationships and their within-subgroup relationships were within a range that was reasonable for full-sibs (compared to the most reliable full-sib families where we could confirm that both parents were correct, Figure II). On this basis, we split the originally presumed families into sub-groups that we identified as full-sib families. In total we obtained eight full-sib families and one half-sib family that consisted of five full-sib families. We therefore set halfsib=1 when running the "ParentCall2" module in LepMap3 to use this information. In cases where we could not identify the correct parents, we assigned placeholder parental IDs (e.g. "mother_fam8.1"). LepMap3 can construct linkage maps even without complete parental data, but a pedigree in the right format is required to identify families. Individuals that did not show close relationships with other F2 or F1 individuals, or could not be classified without doubt, were excluded. The final numbers of F2 individuals per family are given in Table II.

The presumed family 8.1 consisted of two groups of closely related individuals (Figure III). Combining relatedness among offspring with relationship data to the presumed mother showed that one of these full-sib groups was indeed the offspring of the planned mother while the other was not. Individuals within this second group were not related to any of the candidate parents. We excluded them.

We proceeded in the same way with the other potential families, shown in Fig III-VII. Table II shows how the presumed families were split into subgroups.

Family 8-2 consisted of two clusters (Figure IV). One of the clusters was sired by the planned father whereas the other, much smaller cluster was not. Genetic data for the potential mother was not available. Individuals not closely related to the planned father were excluded.

Family 8-3 (Figure V) included a cluster where both of the presumed parents were correct (family 83.1). Another cluster showed an intermediate relationship to the presumed parents, which can occur if the true parents were related to the presumed ones (avuncular relationship). The relationship between individuals within this cluster was within the range (Figure II) that we observed for the family 83.1, which we knew to be a true full-sib family since both parents were correct. We therefore decided to classify this cluster (family 83.2) as

a full-sib family and assigned place holder parental IDs (e.g. “Mother_83.2”) as their parent IDs in the pedigree. Individuals in the other clusters were excluded, because they either did not show a close relationship to presumed parents making it therefore unclear whether they were descendants of the originally crossed F1-families or the potential full-sib family was too small.

Family 9-1 (Figure VI) consisted of two main clusters. Most of the individuals were offspring of the presumed mother. Genetic data for the father was not available. Two individuals that were not offspring of the presumed mother were excluded. We observed that the relationship to the mother of individuals of one cluster was higher (fam 91.1), presumably due to inbreeding when the mother mated with her brother before the planned cross took place. We divided Family 9-1 therefore into two full-sib families and assigned placeholder IDs for the two fathers.

Family 9-2 consisted of three clusters (Figure VII). Individuals of the first cluster (three individuals) were unrelated to both presumed parents and were excluded. Another cluster consisted of individuals that were indeed offspring of the presumed parents and were thus classified as a full-sib family. Individuals of the last cluster were offspring of the presumed mother but unrelated to the potential father. Relationships within this cluster were high (Figure II). We therefore decided to classify it as a full-sib family (fam92.1).

Family 9-3 (Figure VIII) included a few individuals that were unrelated to the presumed parents and were thus excluded. The presumed father was correct for all remaining individuals. These individuals were related to the presumed mother, but not as closely as expected for a parent-offspring relationship (Figure VIII B, please note the difference in relatedness to candidate father and mother). It seemed likely that they were offspring of another female of the same F1 family. Relationships within this large cluster showed a high variation (Figure II). When we first classified them as full-sibs we obtained linkage maps that were inflated. We therefore thought it likely that they represented offspring of several females and we therefore split the large cluster into five smaller clusters, each of them representing a full-sib family. Relationships within these clusters were then within the range of the other full-sib families (Figure II) and we did not obtain inflated linkage maps.

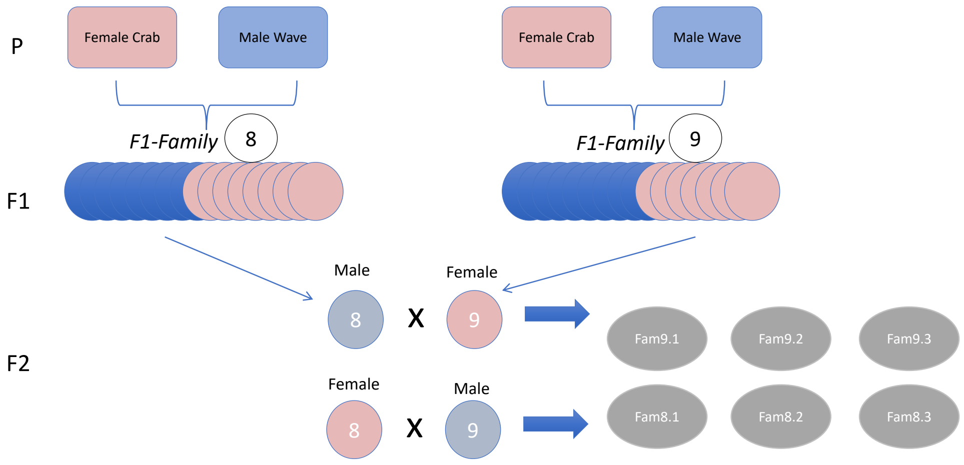

Figure I Crossing design to produce F2- individuals for QTL analysis. Parental individuals (adapted to crab predation and wave exposure) were collected at the Swedish West Coast at Ängklåvebukten.

Table I Genetic data for potential parents of F2-families available

	FAM_8-1	FAM_8-2	FAM_8-3	FAM_9-1	FAM_9-2	FAM_9-3
Mother	yes	no	yes	yes	yes	yes
Father	no	yes	yes	no	yes	yes

Table II: Number of individuals included for each of the identified full-sib families. The full-sib families marked with * are half-sib families to each other

	identified fullsib-families	N	Mother	Father
FAM_8-1	fam81-1	42	I23015	unknown
FAM_8-2	fam82-1	41	unknown	I23006
FAM_8-3	fam83-1	43	I23004	I23003
	fam83-2	13	unknown	unknown
FAM_9-1	fam91-1	35	I23007	unknown
	fam91-2	23	I23007	unknown
FAM_9-2	fam92-1	17	I23009	unknown
	fam92-2	41	I23009	I23010
FAM_9-3	fam93-1*	25	unknown	I23014
	fam93-2*	32	unknown	I23014
	fam93-3*	23	unknown	I23014
	fam93-4*	29	unknown	I23014
	fam93-5*	22	unknown	I23014

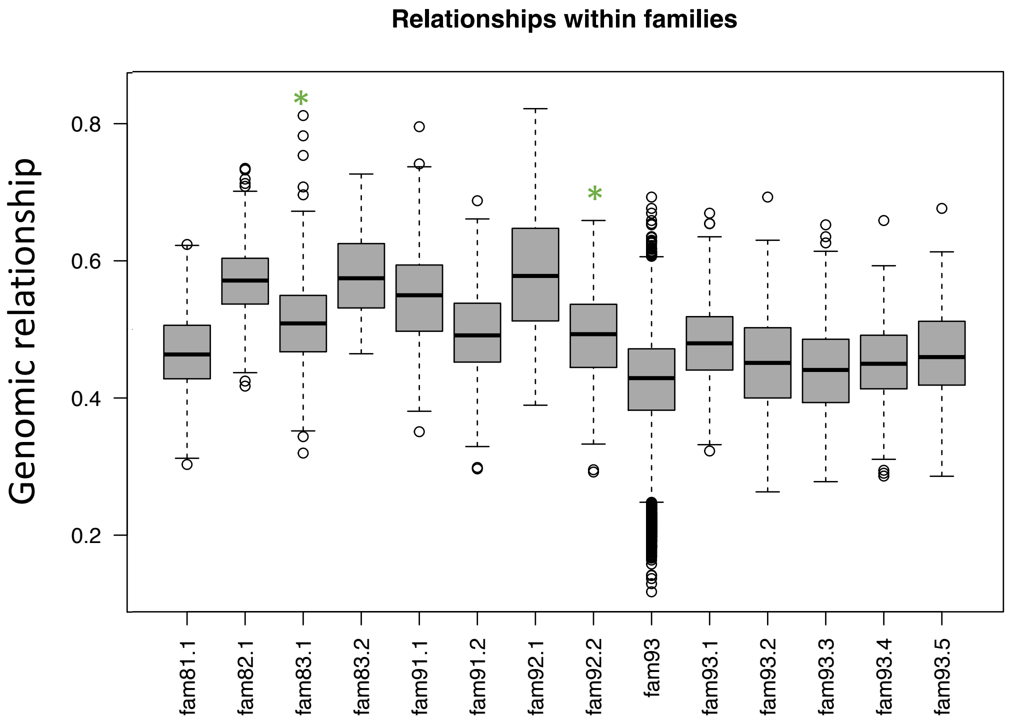

Figure II: Relationships between individuals within the identified full-sib clusters and halfsib-family fam93. Green stars indicate those families where we could confirm that both presumed parents were correct and thus represent the most reliable ones.

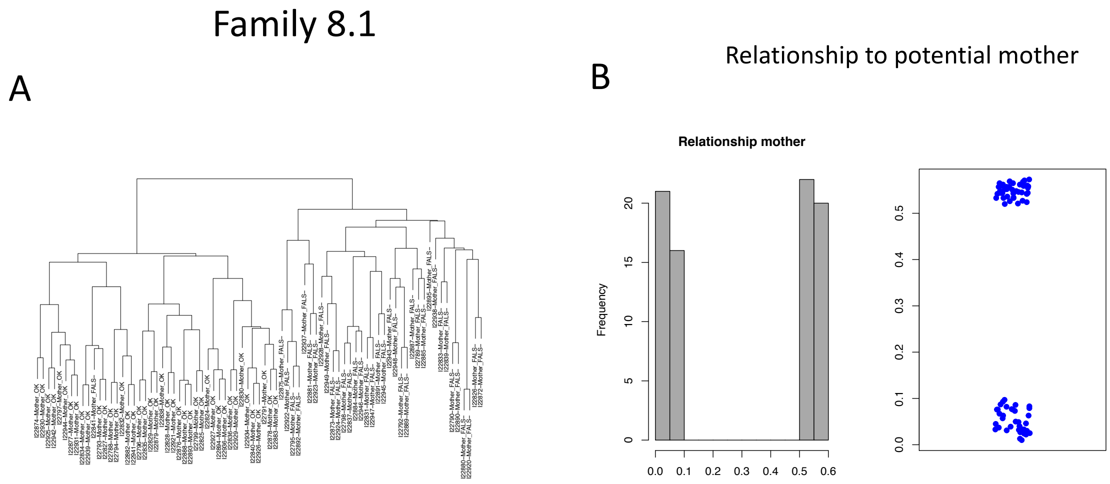

Figure III Relationship within the presumed family 8.1. Relationship between individuals based on additive genetic relationships (A) and the relationship to presumed mother (B). Labeling in (A) as ´OK´ or ´FALSE' is based

on information whether the presumed mother was correct (relationship >0.4 obtained from IBD module in LepMap which corresponds roughly to 0.2 in genomic relationships using the method proposed by VanRaden (2008) as implemented in the Rpackage “AGHmatrix”(Amadeu et al. 2016)) or false (< 0.2 IBD module LepMap; < 0 genomic relationships). Genetic data for the potential father was not available.

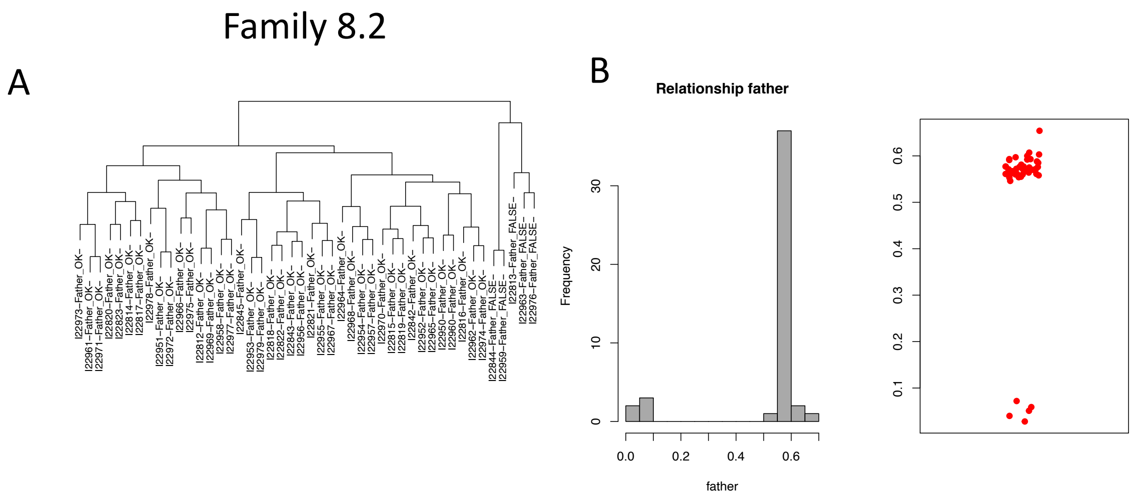

Figure IV Relationship within the presumed family 8.2. Relationship between individuals (A) and the relationship to presumed father (B). Labeling in (A) consists of information whether the presumed father was correct (relationship >0.4) or false (< 0.2). Genetic data for the potential mother was not available.

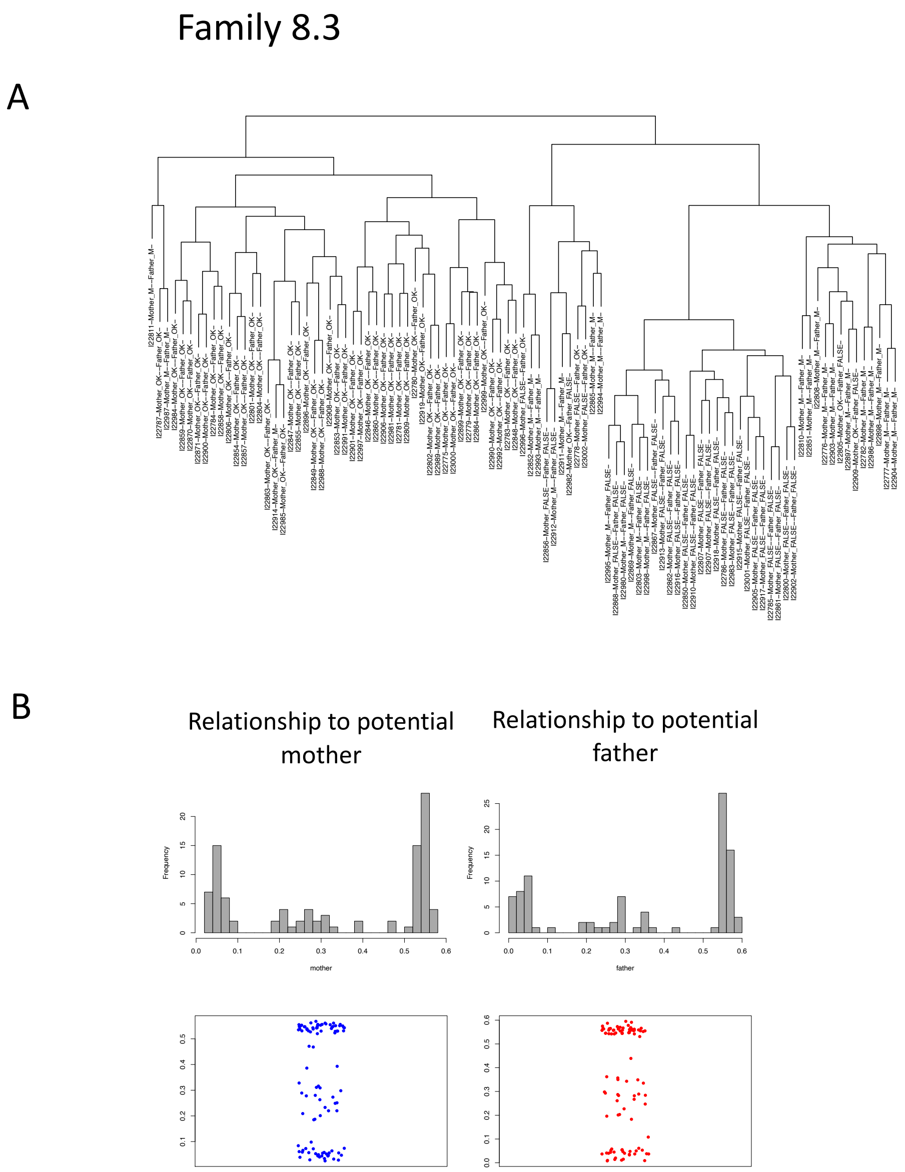

Figure V Relationship within the presumed family 8.3. Relationship between individuals (A) and the relationship to presumed parents (B). Labeling in (A) consists of information whether the presumed parents were correct (relationship >0.4) or false (< 0.2). M indicates an intermediate relationship (0.2-0.4), which can occur if the true parent was a fullsib to the presumed one.

A Family 9.1

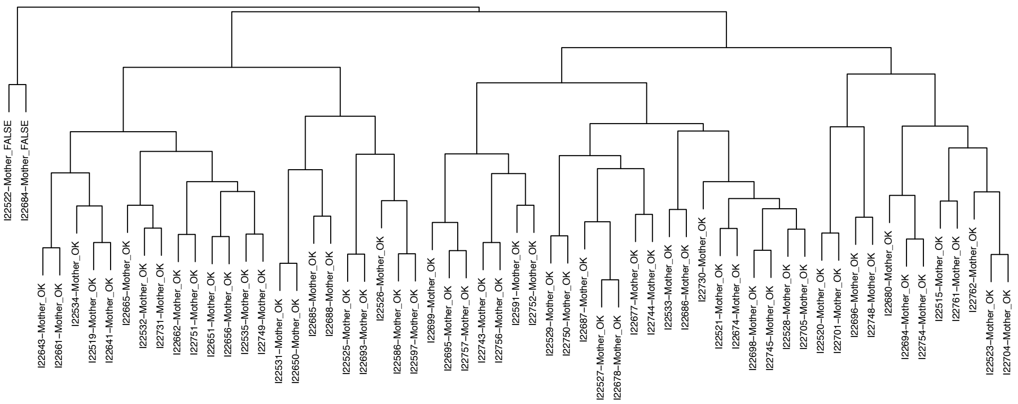

B

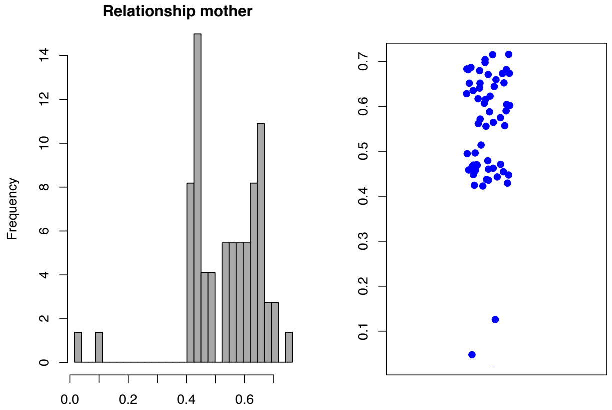

Figure VI Relationship within the presumed family 9.1. Relationship between individuals (A) and the relationship to presumed mother(B). Labeling in (A) consists of information whether the presumed mother was correct (relationship >0.4) or false (< 0.2). The two clusters represent maternal half-sib families. One of these families is likely to be the result of inbreeding (mating of the mother with her brother) as is indicated by elevated relationship with the mother. There was no genetic data for the presumed father available.

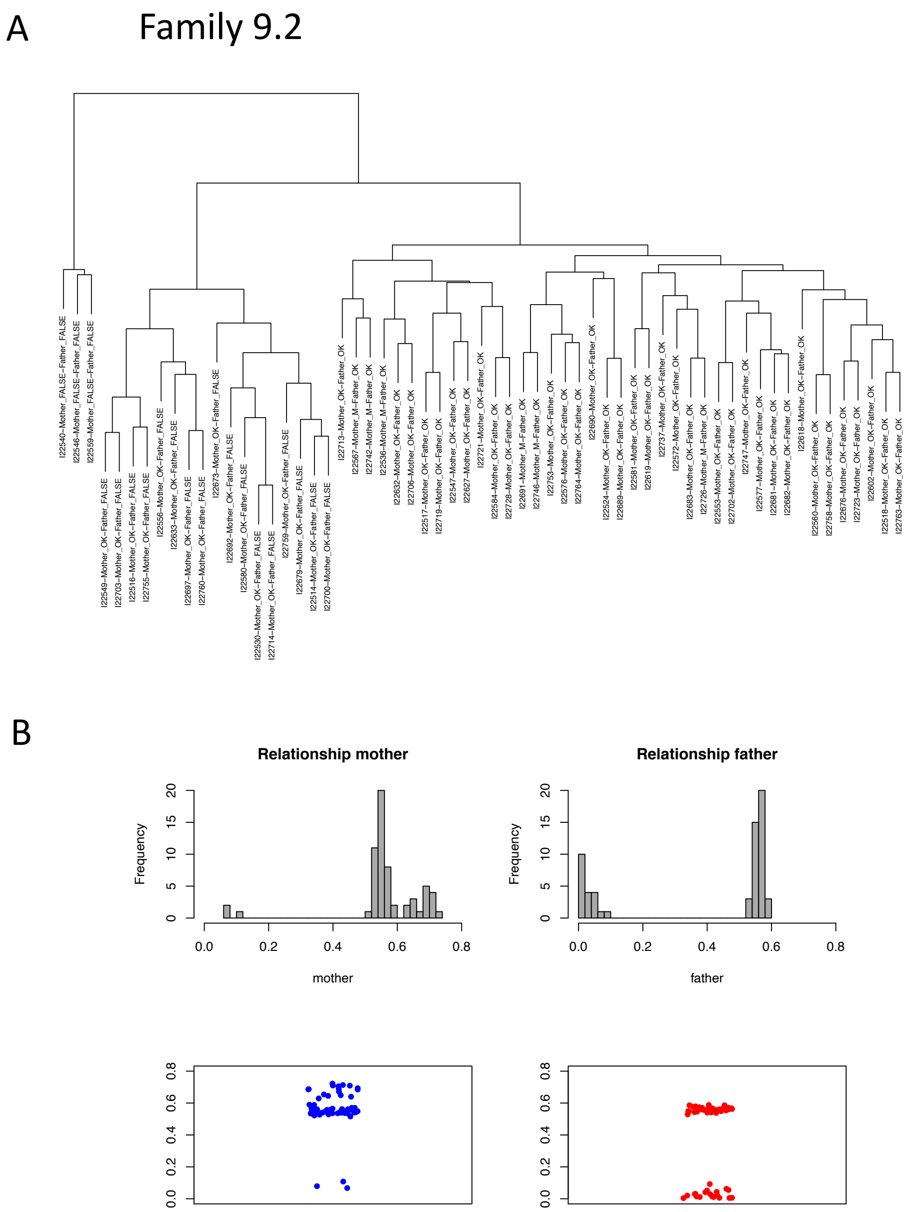

Figure VII Relationship within the presumed family 9.2. Relationship between individuals (A) and the relationship to presumed parents(B). Labeling in (A) consists of information whether the presumed parents were correct (relationship >0.4) or false (< 0.2). Two of the clusters represent maternal half-sib families. One of these families is likely to be the result of inbreeding (mating of the mother with her brother) as is indicated by elevated relationship with the mother.

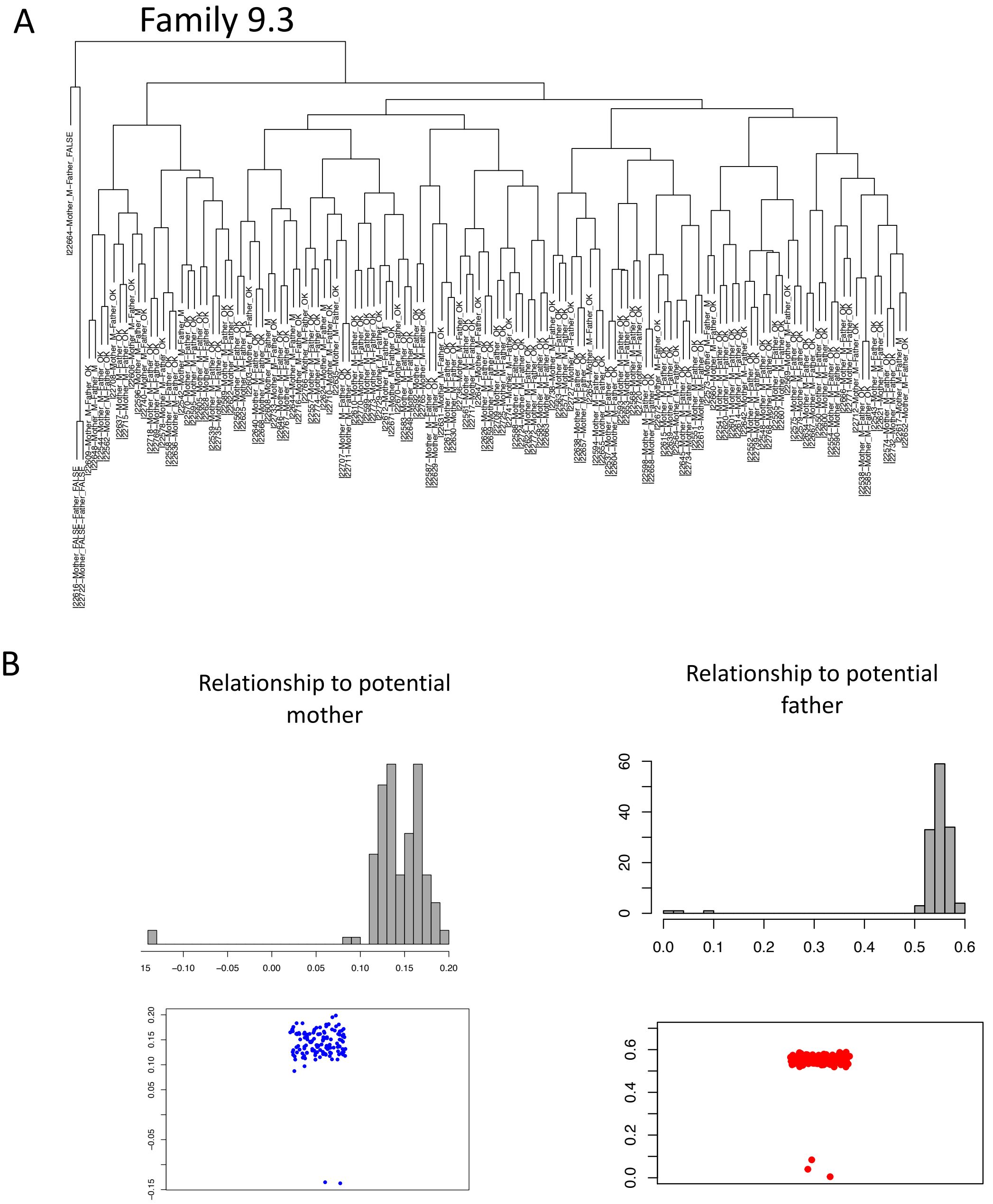

Figure VIII Relationship within the presumed family 9.3. Relationship between individuals (A) and the relationship to presumed parents (B). Labeling in (A) consists of information whether the presumed parents were correct (relationship >0.4) or false (< 0.2). M indicates an intermediate relationship (0.2-0.4), which can occur if the true parent was a fullsib to the presumed one. Here, the true mothers of the different clusters are likely to be sister of the presumed one.

References

Amadeu, R. R., C. Cellon, J. W. Olmstead, A. A. F. Garcia, M. F. R. Resende, and P. R. Muñoz. 2016. AGHmatrix: R package to construct relationship matrices for autotetraploid and diploid species: A blueberry example. Plant Genome 9.

Andrews, S. 2010. FastQC: A quality control tool for high throughput sequence data. Retrieved from https://www.bioinformatics.babraham.

Bolger, A. M., M. Lohse, and B. Usadel. 2014. Trimmomatic: A flexible trimmer for Illumina sequence data. Bioinformatics 30:2114–2120.

Danecek, P., A. Auton, G. Abecasis, C. A. Albers, E. Banks, M. A. DePristo, R. E. Handsaker, G. Lunter, G. T. Marth, S. T. Sherry, G. McVean, and R. Durbin. 2011. The variant call format and VCFtools. Bioinformatics 27:2156–2158.

Depristo, M. A., E. Banks, R. Poplin, K. V. Garimella, J. R. Maguire, C. Hartl, A. A. Philippakis, G. Del Angel, M. A. Rivas, M. Hanna, A. McKenna, T. J. Fennell, A. M. Kernytsky, A. Y. Sivachenko, K. Cibulskis, S. B. Gabriel, D. Altshuler, and M. J. Daly. 2011. A framework for variation discovery and genotyping using next-generation DNA sequencing data. Nat. Genet. 43:491–501.

Faria, R., P. Chaube, H. E. Morales, T. Larsson, A. R. Lemmon, E. M. Lemmon, M. Rafajlović, M. Panova, M. Ravinet, K. Johannesson, A. M. Westram, and R. K. Butlin. 2019. Multiple chromosomal rearrangements in a hybrid zone between *Littorina saxatilis* ecotypes. Mol. Ecol. 28:1375–1393.

Li, H., and R. Durbin. 2009. Fast and accurate short read alignment with Burrows-Wheeler transform. Bioinformatics 25:1754–1760.

Luu, K., E. Bazin, and M. G. B. Blum. 2017. pcadapt: an R package to perform genome scans for selection based on principal component analysis. Mol. Ecol. Resour. 17:67–77.

Rastas, P. 2017. Lep-MAP3: Robust linkage mapping even for low-coverage whole genome sequencing data. Bioinformatics 33:3726–3732.

VanRaden, P. M. 2008. Efficient methods to compute genomic predictions. J. Dairy Sci. 91:4414–4423.

Westram, A. M., M. Rafajlović, P. Chaube, R. Faria, T. Larsson, M. Panova, M. Ravinet, A. Blomberg, B. Mehlig, K. Johannesson, and R. Butlin. 2018. Clines on the seashore: The genomic architecture underlying rapid divergence in the face of gene flow. Evol. Lett. 2:297–309.
